# Safety and Efficacy of Camostat Mesylate for Covid-19: a systematic review and Meta-analysis of Randomized controlled trials

**DOI:** 10.1186/s12879-024-09468-w

**Published:** 2024-07-19

**Authors:** Ubaid Khan, Muhammad Mubariz, Yehya Khlidj, Muhammad Moiz Nasir, Shrouk Ramadan, Fatima Saeed, Aiman Muhammad, Mohamed Abuelazm

**Affiliations:** 1 King Edward Medical University, Lahore, Pakistan; 2 Akhtar Saeed Medical and Dental College, Lahore, Pakistan; 3 Faculty of medicine, Algiers University, Alger Centre, Algeria; 4 Dow University of health science, Karachi, Pakistan; 5 Faculty of medicine, Ain Shams University, Cairo, Egypt; 6 Khyber Girls Medical College, Peshawar, Pakistan; 7 Faculty of Medicine, Tanta University, Tanta, Egypt

**Keywords:** Camostat Mesylate, Covid-19, Pandemic, SARS-CoV-2, Review, Analysis

## Abstract

**Background:**

Camostat mesylate, an oral serine protease inhibitor, is a powerful TMPRSS2 inhibitor and has been reported as a possible antiviral treatment against COVID-19. Therefore, we aim to assess the safety and efficacy of camostat mesylate for COVID-19 treatment.

**Methods:**

A systematic review and meta-analysis synthesizing randomized controlled trials from PubMed, Scopus, Embase, Cochrane, Web of Science, clinical trials.gov, and medrxiv until June 2023. The outcomes were pooled using Mean difference (MD) for continuous outcomes and risk ratio (RR) for dichotomous outcomes. The protocol is registered in PROSPERO with ID CRD42023439633.

**Results:**

Nine RCTs, including 1,623 patients, were included in this analysis. There was no difference between camostat mesylate and placebo in producing negative PCR test results at 1–7 days (RR: 0.76, 95% CI: [0.54, 1.06] *P* = 0.1), 8–14 days (RR: 1.02, 95% CI: [0.84, 1.23] *P* = 0.87), or 15–21 days (RR: 0.99, 95% CI: [0.82, 1.19] *P* = 0.90); clinical resolution of symptoms at 1–7 days (RR: 0.94 (95% CI: 0.58, 1.53) *P* = 0.81), 8–14 days (RR: 0.91, 95% CI: [0.74, 1.11] *P* = 0.33, ), or 15–21 days (RR: 0.77, 95% CI: [0.40, 1.51] *P* = 0.45); and time to symptom improvement (MD:-0.38 weeks (95% CI: [-1.42, 0.66] *P* = 0.47, I^2^ = 85%).

**Conclusion:**

Camostat mesylate did not improve clinical outcomes in patients with COVID-19, compared to placebo.

**Supplementary Information:**

The online version contains supplementary material available at 10.1186/s12879-024-09468-w.

## Introduction

Coronavirus disease 2019 (COVID-19) is a novel coronavirus that originated in China’s Hubei region and spread throughout the world in late 2019 [1–3]. On March 11th, 2020, the WHO classified COVID-19 as a pandemic. COVID-19 is extremely contagious and has put an enormous burden on healthcare systems around the world. Pharmacological treatment of infected patients is required until herd immunity is acquired by extensive viral outbreaks or an effective prophylactic vaccination, since social distance is not an effective long-term stand-alone method.

Current treatment of COVID-19 is primarily hospital-based and directed at advanced disease, for example with remdesivir with FDA approval based on three pivotal trials [4–7], and corticosteroids such as dexamethasone [8, 9]. Furthermore, Monoclonal antibodies can be used in the outpatient setting but they are expensive, logistically challenging to administer, and have variable degrees of efficacy due to viral variants [9].

Despite the recent progress of antiviral drugs, further therapeutic alternatives are still required, especially for post-exposure prophylaxis and COVID-19 early treatment in outpatient settings. New pharmaceutical targets have been suggested as viable options for antiviral drugs against COVID-19. To clarify, viral replication and disease progression can be effectively stopped by blocking viral host cell entry. Previous experimental data [10–12] show that the SARS-CoV-2 spike (S) protein binds to target cells via the host cell factors angiotensin-converting enzyme 2 (ACE-2) and that S protein cleavage by the host cell surface trans-membrane protease serine 2 (TMPRSS2) allow entry into target cells.

Camostat mesylate has been used in clinical settings to treat pancreatitis and reflux esophagitis for over two decades [11–13]. Camostat mesylate molecules inhibit TMPRSS2 priming of S protein, a process that has been demonstrated to be both essential and sufficient for viral entry into respiratory epithelial cells [11, 12]. Also, COVID-19 infection of primary human lung epithelial cells was demonstrated to be inhibited by camostat mesylate. Camostat mesylate is a prodrug that, upon entering the bloodstream, rapidly converts to the pharmacologically active metabolite FOY-251, which inhibits TMPRSS2. FOY-251 has an EC50 of 178 nM against SARS-CoV-2 infection in Calu-3 lung cell culture [11]. Moreover, even at high dosages, it has few, mild adverse effects and is readily produced at low costs. Hence, camostat mesylate was predicted to be a good candidate for the treatment of COVID-19. This systematic review and meta-analysis aims to synthesize evidence from randomized controlled trials (RCTs), investigating the efficacy and safety of camostat mesylate for COVID-19 treatment.

## Methodology

### Protocol Registration

The Preferred Reporting Items for Meta-Analyses according to (PRISMA) guidelines [14] were followed for this meta-analysis. Our protocol was prospectively registered in the International Prospective Register of Systematic Reviews (PROSPERO) with ID CRD42023439633.

### Data source and search strategy

An electronic search of PubMed, Scopus, Embase, Cochrane, Web of Science, clinical trials.gov, and medrxiv was conducted from inception to June 2023 without any search restrictions. In addition, references from any retrieved trials were screened manually to identify potentially relevant articles. Further details regarding data source and search strategy are given in (Table S1).

### Eligibility criteria

A PICO criterion was used to include RCTs: population (P): patients with COVID-19 regardless of the disease severity; intervention (I): camostat mesylate; control (C): placebo with or without the standard of care; and outcomes (O): primary outcomes of this review were the efficacy outcomes: all-cause mortality, PCR negative, clinical resolution of symptoms, time to symptom improvement, hospitalization duration, and intensive care unit (ICU) admission or mechanical ventilation. The secondary outcomes included safety outcomes: any adverse events, any serious adverse, elevated liver enzymes, and specific safety events.

### Study selection

Three reviewers (A.I., S.R., & M.M.) independently screened the studies using Covidence [15] after duplicates were screened and removed automatically. The remaining studies were carefully assessed in accordance with the eligibility criteria. All studies were initially short-listed based on title and abstract, and subsequently, full-length articles were reviewed. Any discrepancies and conflicts between the selected studies were resolved by a U.K.

### Data extraction

Four reviewers (A.I., S.R., M.M., & M.M.N.) extracted data independently, including baseline, efficacy, and safety data. Baseline data included number of participants in each, mean age, gender, mean body mass index (BMI), mean duration of symptoms, ordinal severity score, and comorbidity data. Efficacy data was recorded in terms of number of patients with negative PCR (at 1–7 days, 8–14 days, and 15–21 days or more), clinical resolution of symptoms (at 1–7 days, 8–14 days, and 15–21 days or more), time to improvement in symptoms, viral load at the end of follow up, duration of hospitalization, all-cause mortality, and ICU admission or mechanical ventilation. Safety data included the incidence of any adverse event, any serious adverse event, and specific adverse events. Conflicts were solved by mutual discussion between reviewers.

### Risk of Bias and Certainty of evidence

Four reviewers (A.I., S.R., M.M., & F.S) independently assessed the quality of included studies using the modified Cochrane Collaboration’s risk of bias tool for randomized controlled trials [16] Conflicts were solved by mutual discussion between reviewers.

To appraise the quality of evidence, two reviewers (M.A. and U.K.) utilized the Grading of Recommendations Assessment, Development, and Evaluation (GRADE) guidelines [17, 18]. We considered inconsistency, imprecision, indirectness, publication bias, and risk of bias. The evaluation was carried out for each outcome, and the decisions made were justified and documented. Any discrepancies were settled through discussion.

We followed the confidence interval cutoffs provided by Cochrane consumers and communication “how to grade?” guidelines [19].

### Statistical analysis

RevMan (version 5.3; Copenhagen: The Nordic Cochrane Centre, The Cochrane Collaboration, 2014) was used for all statistical analyses [20]. The results from trials were presented as risk ratios (RR) for dichotomous outcomes and mean difference (MD) for continuous outcomes with a 95% confidence interval (CI) and were pooled using a fixed-effects model in case of homogenous data and random effects model in case of heterogeneous data. According to the Cochrane Handbook (chapter nine) [21]., heterogeneity was considered significant if the alpha value of the Chi-square test is below 0.1, while the interpretation of the I-square test is as follows: (0–40%) not significant, (30–60%) moderate heterogeneity, (50–90%) substantial heterogeneity, and (75–100%) considerable heterogeneity.

## Results

### Search results and study characteristics

**Fig. 1:**
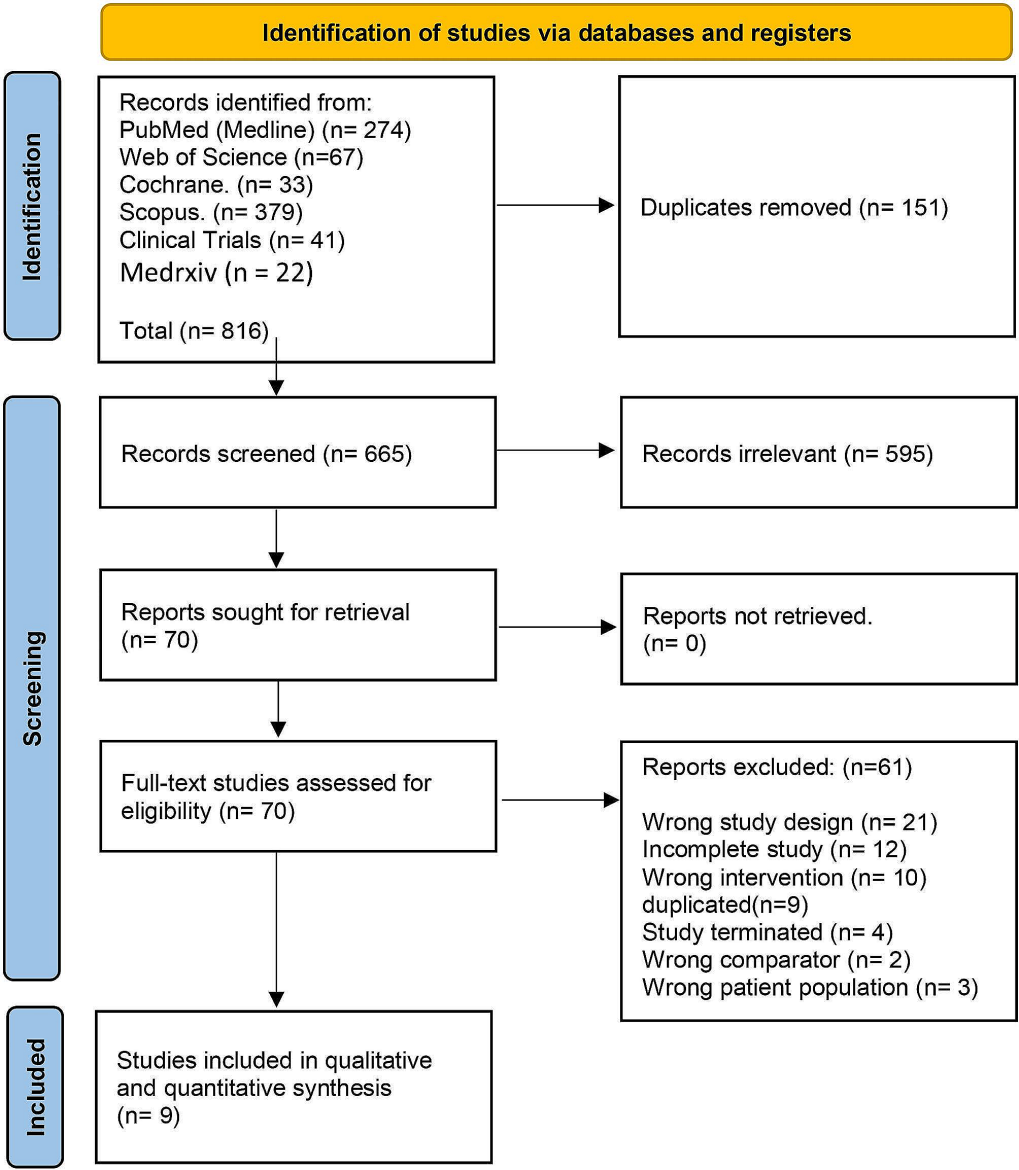
PRISMA flow chart of the screening process

The initial literature search yielded 816 studies after the removal of duplicate (*n* = 151) and irrelevant studies (*n* = 656), leaving nine RCTs for inclusion in the final quantitative and qualitative analysis. Out of total, 63 studies were excluded in full text screening with reason of exclusion mentioned in (Table S2). Finally, nine studies were included in the final analysis. Further details can be obtained from the PRISMA flowchart in (Fig. 1**)**.

### Included studies characteristics

Nine RCTs [22–30] were included in the final analysis with a total of 1,623 participants (*n* = 912 in the camostat mesylate group and *n* = 711 in the placebo group), with 52.7% of the patients being male. Most of the studies were conducted in the USA (*n* = 4), followed by an equal number of trials from Sweden, Austria, Japan, Denmark, Belgium, and South Korea. Camostat mesylate and placebo were given as oral tablets. The mean duration of follow-up was 2.8 weeks. The definition and criteria for serious adverse events were different in each article so we have explained it in Table S3 to make it clear. Further information about baseline study and patient characteristics are available in (Tables 1, 2), respectively.

**Table 1:** Summary characteristics of the included trials

Study ID	Study Design	Country	COVID-19 Severity	Total Participants	Camostat Mesylate				Control	Primary Outcome	Method of viral eradication assessment	Time of viral eradication assessment	Clinical resolution assessment criteria	Follow-Up Duration
					Form	Dose	Times of administration/Day	TTT Duration						
**Chupp et al. 2022** [22]	Phase II, Double-Blind, RCT	USA	Outpatient Mild	70	Oral tablet	200 mg	Four	7 days	Placebo	Reduction in viral load.	Nasopharyngeal swab (RT-PCR)	0,2,4,6	N/A	4 weeks
**Karolyi et al. 2022** [29]	Multi-Center open-label RCT	Austria	Hospitalized moderate to severe	201	Oral tablet	100 mg	Two	N/A	Lopinavir/ritonavir	Time to sustained clinical improvement (≥ 48 h)	N/A	N/A	Seven-category ordinal scale	4 weeks
**Jilg et al. 2023** [23]	Phase II RCT	USA	Hospitalized Mild to moderate	216	Oral tablet	200 mg	Two	7 days	Placebo	Reduction in viral load	Nasopharyngeal swab (RT-PCR)	3,7,14	Likert scale	4 weeks
**Kim et al. 2022** [28]	Double-blinded, Phase II RCT	South Korea	Mild to moderate	342	Oral tablet	N/A	N/A	14 days	Placebo	Time to clinical improvement through 14 days	N/A	7	Likert scale	2 weeks
**Tobback et al. 2022** [24]	Phase II RCT	Belgium	Outpatient Mild	90	Oral tablet	300 mg	Three	5 days	Placebo	Reduction in viral load	Nasopharyngeal swab (RT-PCR)	1,5,10	Likert scale	5 days
**Gunst et al. 2021** [26]	Double-blinded RCT	Denmark & Sweden	Hospitalized moderate to severe	205	Oral tablet	200 mg	Three	5 days	Placebo	Time to clinical improvement	Oropharyngeal swabs for all patients and blood samples for some patients	Baseline and day 5	N/A	4 weeks
**Kinoshita et al. 2022** [25]	Mult-center, double-blinded, RCT”	Japan	Mild to moderate	155	Oral tablet	600 mg	Three	14 days	Placebo	Time to first two consecutive negative SARS-CoV-2 test at hospitals local laboratory.	Nasopharyngeal swab (RT-PCR)	Daily tests were performed	N/A	2 weeks
**NCT04524663** [30]	Double-blinded, Phase II RCT	USA	Mild to moderate	49	Oral tablet	N/A	NA	10 days	Standard supportive care	Time from randomization to first two consecutive negative PCR test results	Nasopharyngeal swab (RT-PCR)	N/A	N/A	4 weeks
**NCT04583592** [27]	Quadruple-blinded RCT	USA	Mild to moderate	295	Oral tablet	200 mg	Four	14 days	Placebo	Disease progression at day 28	N/A	N/A	N/A	N/A

RCT: Randomized controlled trial, USA: United States of America; N/A: not available

**Table 2:** Baseline characteristics of the participants

Study ID	Number of patients in each group		Age (Years) Mean (SD)		Gender (Male) *N*. (%)		Body mass index, Mean (SD)		Duration of symptoms, Mean (SD)		Ordinal Severity Score *N*. (%)						Comorbidities *N*. (%)											
	CM	Control	CM	Control	CM	Control	CM	Control	CM	Control	3		4		5		Smoking		DM		HTN		COPD		Asthma		Cardiovascular disease	
											CM	Control	CM	Control	CM	Control	CM	Control	CM	Control	CM	Control	CM	Control	CM	Control	CM	Control
**Chupp et al. 2022** [22]	35	35	44.1 (14.6)	44.1 (12.0)	22 (62.9)	20 (57.1)	N/A	N/A	41.1 (25.6)	35.8 (22.4)	N/A	N/A	N/A	N/A	N/A	N/A	N/A	N/A	1 (2.9)	3 (8.6)	10 (28.6)	4 (11.4)	1 (2.9)	0 (0.0)	9 (25.7)	7 (20.0)	0 (0.0)	0 (0.0)
**Karolyi et al. 2022** [29]	101	100	56.6 (17.2)	60.7 (12.6)	67 (66)	67 (67)	30.4 (5.6)	30.1 (5.7)	5.34 (3.01)	4.34 (3.76)	15 (15)	20 (20)	64 (63)	59 (59)	22 (22)	21 (21)	N/A	N/A	20 (20)	34 (34)	47 (47)	58 (57)	16 (16)	14 (14)	N/A	N/A	6	5
**Jilg et al. 2023** [23]	109	107	38.3 (15)	38.6 (14.27)	46 (42.2)	52 (48.5)	27.96 (5.55)	27.7 (5.26)	5.66 (2.25)	5.33 (3)	N/A	N/A	N/A	N/A	N/A	N/A	N/A	N/A	N/A	N/A	N/A	N/A	N/A	N/A	N/A	N/A	N/A	N/A
**Kim et al. 2022** [28]	172	170	52.15 (14.55)	50.68 (15.14)	95 (55.23	87 (51.18)	24.95 (3.66)	24.87 (3.76)	N/A	N/A	94 (54.65)	02 (60.00)	N/A	N/A	N/A	N/A	25 (26.04)	25 (27.78)	28 (29.17)	19 (21.11)	50 (52.08)	54 (60.00)	1 (1.04)	2 (2.22)	N/A	N/A	13 (13.54)	11 (12.22)
**Tobback et al. 2022** [24]	61	29	38 (21)	36.6 (22.6)	28 (45.9)	13 (44.8)	23.8 (2.8)	25 (3.9)	1 (83.6)	26 (89.7)	N/A	N/A	N/A	N/A	N/A	N/A	19 (31.1)	9 (31.0)	1 (1.6)	0 (0)	N/A	N/A	N/A	N/A	N/A	N/A	N/A	N/A
**Gunst et al. 2021** [26]	137	68	62.7 (18.0)	63.3 (14.4)	82 (60)	41 (60)	27.8 (5.4)	29.2 (5.2)	NA	NA	47 (34)	22 (32)	81 (59)	39 (57)	9 (07)	7 (10)	NA	NA	21 (15)	14 (21)	50 (36)	21 (31.0)	14 (10)	7 (10)	18 (13)	9 (13)	29 (21)	10 (15)
**Kinoshita et al. 2022** [25]	78	77	55.7 (18.8)	56.1 (18.2)	35 (44.9)	43 (55.8)	24.5 (5.2)	23.9 (3.7)	3.3 (1.2)	3.5 (1.1)	78 (100)	77 (100)	NA	NA	NA	NA	NA	NA	15 (19.2)	12 (15.6)	24 (30.8)	20 (26.0)	11 (14.1)	14 (18.2)	NA	NA	4 (5.1)	4 (5.2)
**NCT04524663** [30]	25	24	37.9 (13.5)	40.5 (14.3)	15 (60)	17 (70.8)	28.7 (6.7)	27.7 (4.1)	4.6 (2.6)	4.4 (1.0)	NA	NA	NA	NA	NA	NA	NA	NA	NA	NA	NA	NA	NA	NA	NA	NA	NA	NA
**NCT04583592** [27]	194	101	NA	NA	86 (44.3)	40 (39.6)	NA	NA	NA	NA	NA	NA	NA	NA	NA	NA	NA	NA	NA	NA	NA	NA	NA	NA	NA	NA	NA	NA

N. Number; SD. Standard Deviation; CM. Camostat mesylate

### Risk of Bias and Certainty of evidence

**Fig. 2:**
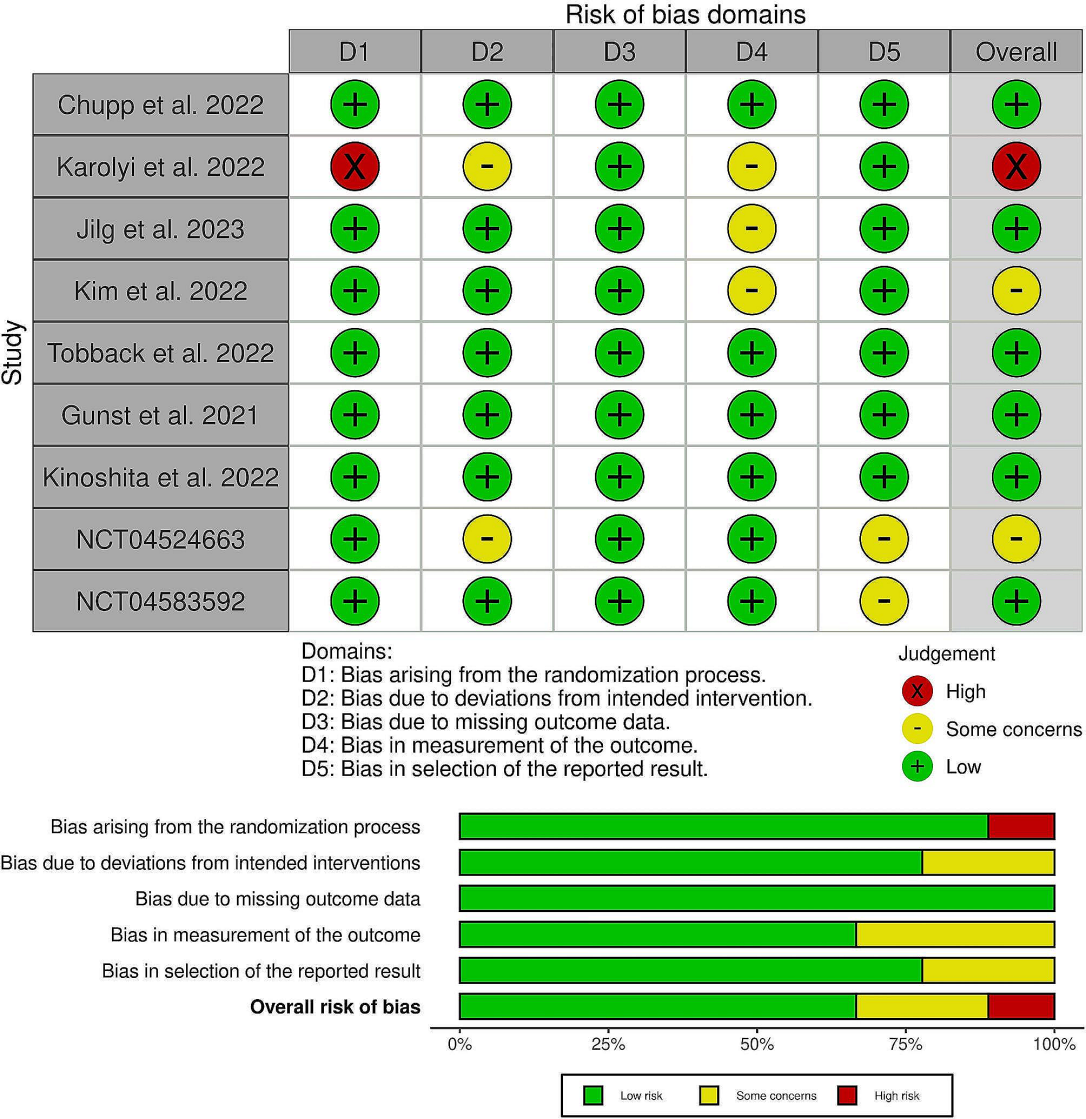
Quality assessment of risk of bias in the included trials. The upper panel presents a schematic representation of risks (low = red, unclear = yellow, and high = red) for specific types of biases of each of the studies in the review. The lower panel presents risks (low = red, unclear = yellow, and high = red) for the subtypes of biases of the combination of studies included in this review

After a careful assessment using the Cochrane ROB 2.0 tool, six RCTs were concluded as having a low risk of bias [22–27], two showing some concerns [28, 30], and one with a high risk of bias [29].(Fig. 2). Certainty of evidence is demonstrated in detail in a GRADE evidence profile (Table 3). The details of all the domains which are assessed are mentioned in (Table S4-S12).

**Table 3:** GRADE evidence profile

Certainty assessment							Summary of findings				
Participants (studies) Follow-up	Risk of bias	Inconsistency	Indirectness	Imprecision	Publication bias	Overall certainty of evidence	Study event rates (%)		Relative effect (95% CI)	Anticipated absolute effects	
							With Placebo	With Camostat Mesylate		Risk with Placebo	Risk difference with Camostat Mesylate
**All-cause mortality**											
674 (5 RCTs)	very serious^a^	not serious	very serious^b^	very serious^c^	none	⨁◯◯◯ Very low	19/301 (6.3%)	14/373 (3.8%)	**RR 0.55** (0.27 to 1.10)	63 per 1,000	**28 fewer per 1,000** (from 46 fewer to 6 more)
**PCR Negative − 1–7 days**											
941 (5 RCTs)	not serious	not serious	not serious	very serious^c^	none	⨁⨁◯◯ Low	56/426 (13.1%)	59/515 (11.5%)	**RR 0.77** (0.55 to 1.07)	131 per 1,000	**30 fewer per 1,000** (from 59 fewer to 9 more)
**PCR Negative − 8–14 days**											
773 (5 RCTs)	not serious	not serious	not serious	serious^d^	none	⨁⨁⨁◯ Moderate	108/340 (31.8%)	147/433 (33.9%)	**RR 1.03** (0.85 to 1.24)	318 per 1,000	**10 more per 1,000** (from 48 fewer to 76 more)
**PCR Negative − 15–21 days**											
678 (5 RCTs)	not serious	not serious	not serious	not serious	none	⨁⨁⨁⨁ High	122/276 (44.2%)	201/402 (50.0%)	**RR 1.04** (0.91 to 1.20)	442 per 1,000	**18 more per 1,000** (from 40 fewer to 88 more)
**Clinical resolution of symptoms − 1–7 days**											
373 (3 RCTs)	not serious	not serious	not serious	very serious^c^	none	⨁⨁◯◯ Low	61/168 (36.3%)	67/205 (32.7%)	**RR 1.02** (0.78 to 1.34)	363 per 1,000	**7 more per 1,000** (from 80 fewer to 123 more)
**Clinical resolution of symptoms − 8–14 days**											
303 (2 RCTs)	not serious	not serious	not serious	very serious^d^	none	⨁⨁◯◯ Low	73/133 (54.9%)	71/170 (41.8%)	**RR 0.90** (0.73 to 1.10)	549 per 1,000	**55 fewer per 1,000** (from 148 fewer to 55 more)
**Clinical resolution of symptoms − 15–21 days**											
269 (2 RCTs)	not serious	not serious	not serious	very serious^d^	none	⨁⨁◯◯ Low	16/117 (13.7%)	14/152 (9.2%)	**RR 0.77** (0.40 to 1.50)	137 per 1,000	**31 fewer per 1,000** (from 82 fewer to 68 more)
**Time to symptom improvement**											
945 (4 RCTs)	serious^e^	very serious^f^	serious^g^	very serious^h^	none	⨁◯◯◯ Very low	437	508	-		MD **0.38 lower** (1.42 lower to 0.66 higher)
**ICU admission or Mechanical ventilation**											
559 (3 RCTs)	serious^i^	serious^j^	serious^k^	very serious^h^	none	⨁◯◯◯ Very low	21/244 (8.6%)	18/315 (5.7%)	**RR 0.55** (0.20 to 1.53)	86 per 1,000	**39 fewer per 1,000** (from 69 fewer to 46 more)
**Any adverse events**											
1553 (9 RCTs)	not serious	very serious^f^	not serious	not serious	none	⨁⨁◯◯ Low	310/709 (43.7%)	329/844 (39.0%)	**RR 0.94** (0.74 to 1.21)	437 per 1,000	**26 fewer per 1,000** (from 114 fewer to 92 more)
**Any serious adverse events**											
1262 (7 RCTs)	not serious	not serious	not serious	serious^h^	none	⨁⨁⨁◯ Moderate	21/580 (3.6%)	45/682 (6.6%)	**RR 1.77** (1.10 to 2.83)	36 per 1,000	**28 more per 1,000** (from 4 more to 66 more)

CI: confidence interval; MD: mean difference; RR: risk ratio

*Explanations*

a. Karolyi et al. is of high risk of overall bias and constitute 73.7% of the outcome pooled data

b. Karolyi et al. is the only study that used lopinavir/ritonavir as a control, constituting 73.7% of the outcome pooled data

c. Wide confidence interval that does not exclude the risk of appreciable harm/benefit, with a low number of events

d. Low number of events < 300 events

e. Karolyi et al. is of high risk of overall bias and constituting 27.3% of the outcome pooled data

f. I2 > 75%

g. Karolyi et al. is the only study that used lopinavir/ritonavir as a control, constituting 27.3% of the outcome pooled data

h. Wide confidence interval that does not exclude the risk of appreciable harm/benefit

i. Karolyi et al. is of high risk of overall bias and constitute 44.1% of the outcome pooled data

j. I2 > 50%

k. Karolyi et al. is the only study that used lopinavir/ritonavir as a control, constituting 44.1% of the outcome pooled data

### Efficacy outcomes

#### All-cause mortality

**Fig. 3:**
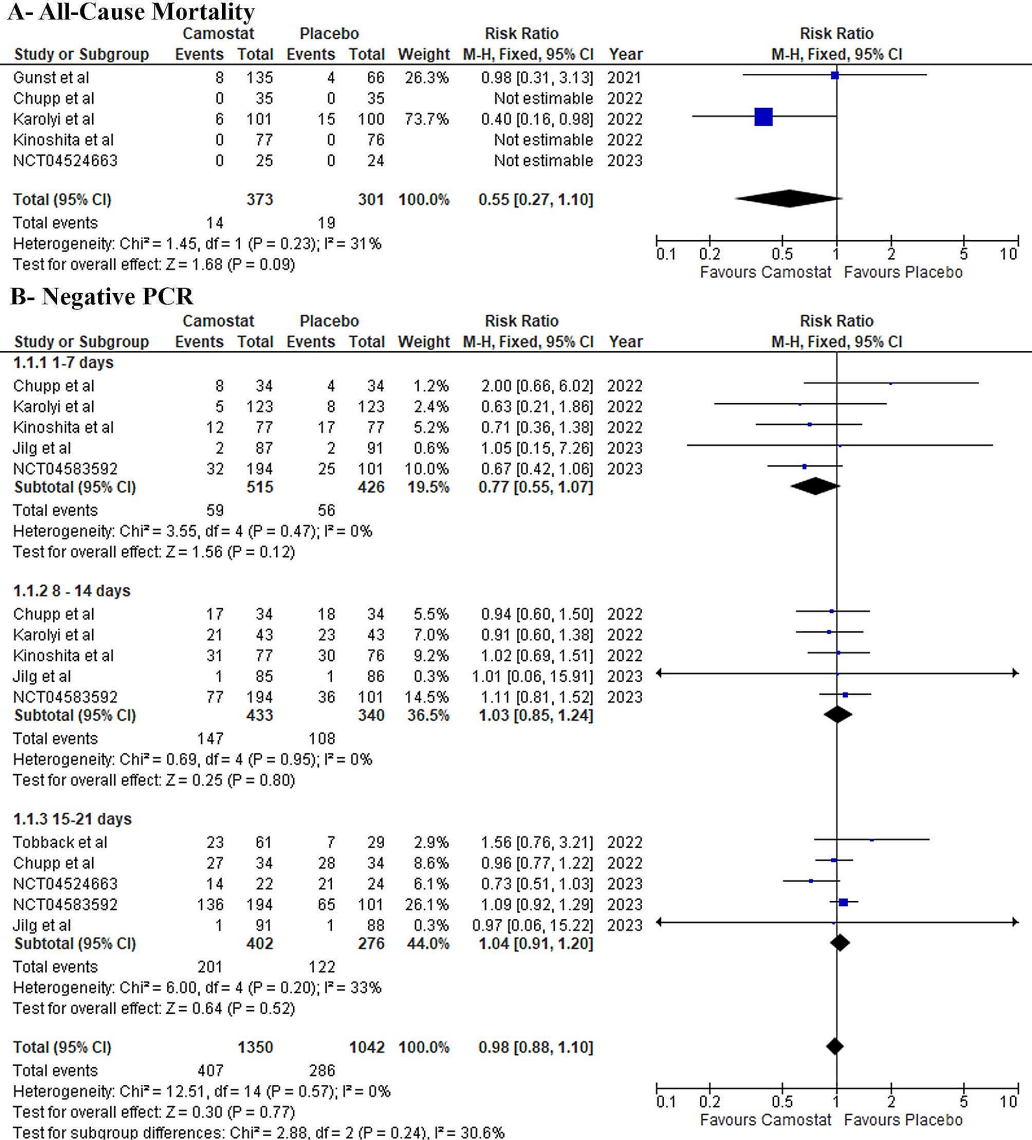
Forest plots of the primary efficacy outcome, RR: risk ratio, MD: mean difference, CI: confidence interval

The analysis showed an insignificant difference between camostat mesylate and the placebo groups, and no significant heterogeneity was observed (RR: 0.55, 95% CI: [0.27, 1.10] *P* = 0.09, I^2^ = 31%) (Fig. 3-A).

#### Negative PCR

There was no difference between both groups at 1–7 days (RR: 0.77, 95% CI: [0.55, 1.07] *P* = 0.12, I^2^ = 0%), 8–14 days (RR: 1.03, 95% CI: [0.85, 1.24] *P* = 0.80, I^2^ = 0%), and 15–21 days (RR: 1.04, 95% CI: [0.91, 1.20] *P* = 0.52, I^2^ = 33%), without any observed significant heterogeneity (Fig. 3-B).

#### Clinical resolution of symptoms

**Fig. 4:**
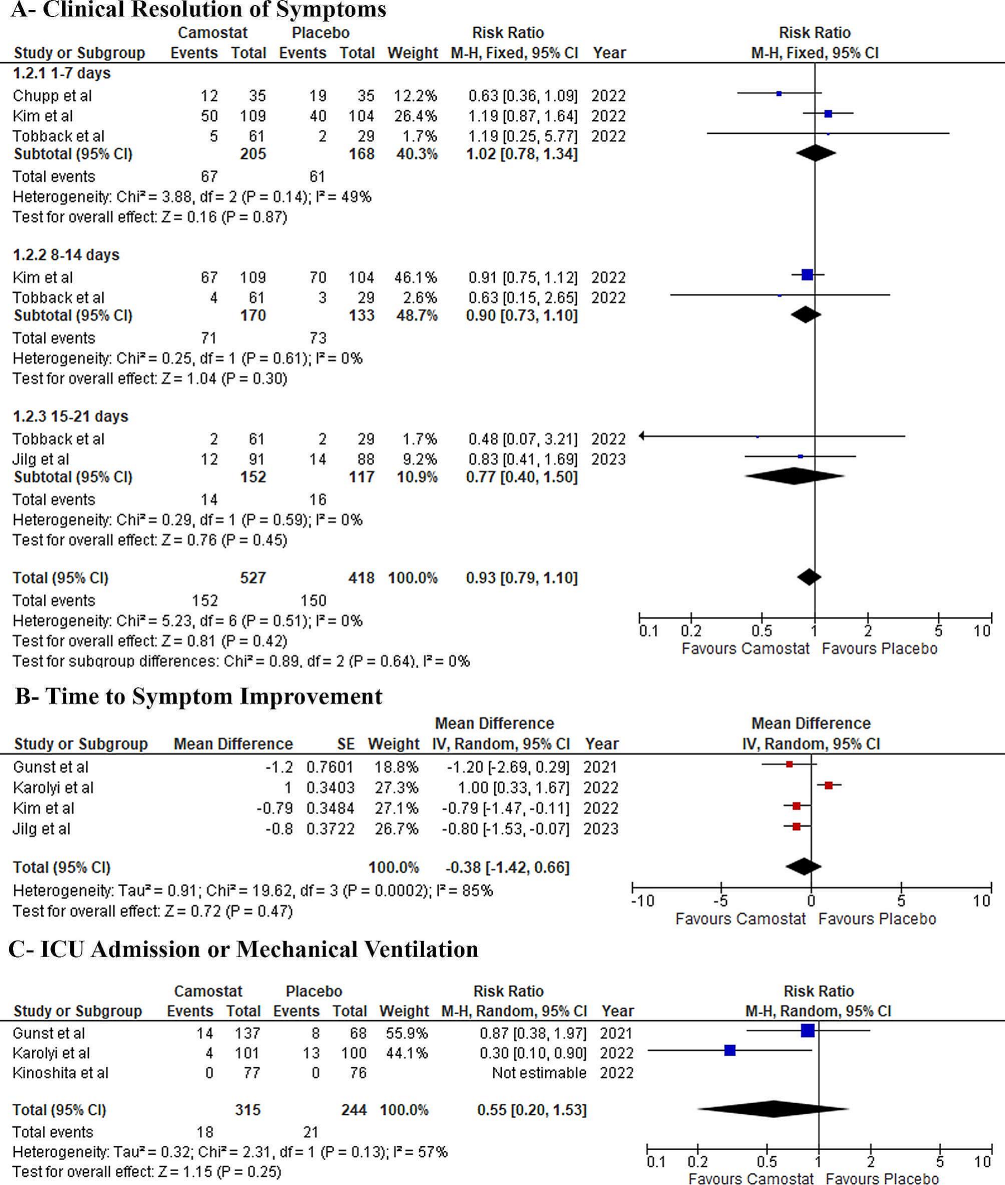
Forest plots of the secondary efficacy outcomes, RR: risk ratio, MD: mean difference, CI: confidence interval

There was no difference between both groups at 1–7 days (RR: 1.02, 95% CI: [0.78, 1.34] *P* = 0.87, I^2^ = 49%), 8–14 days (RR: 0.90, 95% CI: [0.73, 1.10] *P* = 0.30, I^2^ = 0%), and 15–21 days (RR: 0.77, 95% CI: [0.40, 1.50] *P* = 0.45, I^2^ = 0%) without any observed significant heterogeneity (Fig. 4-A).

#### Time to Symptom Improvement

There was no difference between both groups (MD: -0.38 weeks, 95% CI: [-1.42, 0.66] *P* = 0.47, I^2^ = 85%) **(**Fig. 4-B**)**. Significantly high heterogeneity was observed (I^2^ = 85%, *P* = 0.0002) which was resolved by removing Karolyi et al. by leave-one-out sensitivity analysis (I^2^ = 0%, *P* = 0.88) (Table S13).

#### ICU admission or mechanical ventilation

There was no difference between both groups (RR: 0.55, 95% CI: [0.20, 1.53] *P* = 0.25, I^2^ = 57%) (Fig. 4-C). Significant heterogeneity was observed which could not be resolved by a sensitivity analysis (Table S13).

### Safety outcomes

**Fig. 5:**
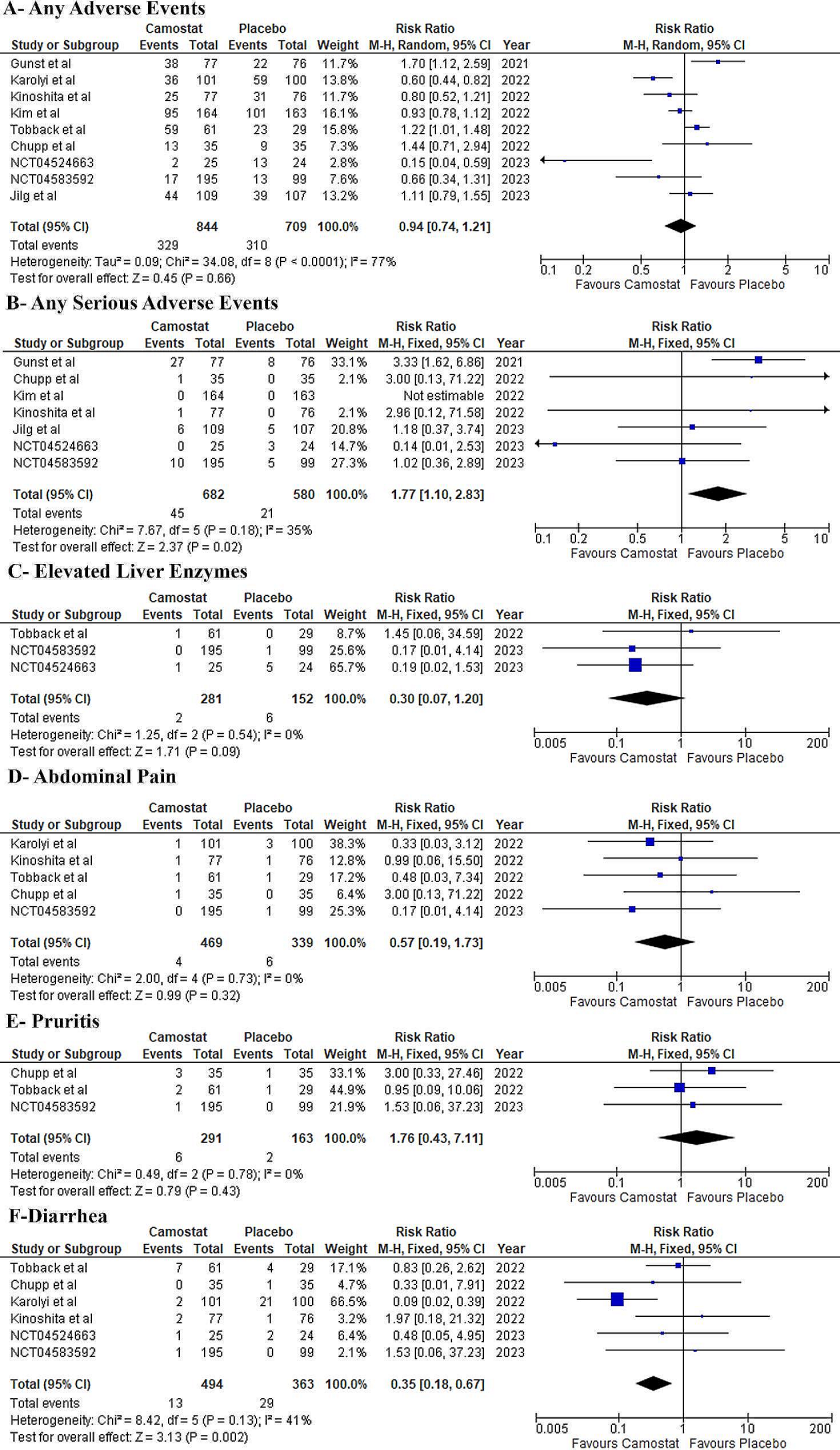
Forest plot of the safety outcomes, RR: risk ratio, CI: confidence interval

There was no difference between both groups regarding the incidence of any adverse events (RR: 0.93, 95% CI: [0.67, 1.29] *P* = 0.66, I^2^ = 80%), elevated liver enzymes (RR: 0.30, 95% CI: [0.07, 1.30] *P* = 0.12, I^2^ = 0%), abdominal pain (RR: 0.57, 95% CI: [0.19, 1.73] *P* = 0.32, I^2^ = 0%), and pruritis (RR: 1.76, 95% CI: [0.43, 7.11] *P* = 0.43, I^2^ = 0%). However, compared to the placebo group, the camostat mesylate group showed a significantly higher risk of any serious adverse events (RR: 1.77, 95% CI: [1.1, 2.83] *P* = 0.02, I^2^ = 35%), and a lower risk of diarrhea (RR: 0.35, 95% CI: [0.18, 0.67] *P* = 0.002, I^2^ = 41%) (Fig. 5). More details about serious adverse events in each study are given in table S13.

Statistically significant heterogeneity was observed in any adverse events outcome (I^2^ = 80%, *p* < 0.0001). A leave-one-out sensitivity analysis was conducted; however, no single study could be found responsible for it (Table S13).

## Discussion

The present systematic review and meta-analysis showed that camostat mesylate is overall ineffective in improving the clinical outcomes of COVID-19 patients while increasing the risk of any serious adverse events. Hence, camostat mesylate exhibited no superiority to placebo in reducing the risk of mortality and ICU admission or mechanical ventilation events. Similarly, it did not accelerate either the clinical recovery (clinical resolution of symptoms and time to symptom improvement) or the viral clearance (time for PCR negativation). Therefore, the current RCTs-based evidence suggests that camostat mesylate when given as monoantiviral therapy for COVID-19 patients may have no particular utility whether in mild, moderate, or severe forms.

Death in patients with acute SARS-CoV-2 infection results from several causes, including multiple organ dysfunction syndrome, nosocomial superinfection (mainly ventilator-associated pneumonia), refractory hypoxemia/pulmonary fibrosis (secondary to extensive lung damage), and fatal ischemic events affecting venous (e.g., pulmonary embolism) or arterial (e.g., stroke and myocardial infarction) circulation [31]. For an antiviral drug to reduce the risk of these events it should prevent the progression to severe COVID-19 and hospitalization by early eradication of infection such as the FDA-approved drugs’ combination nirmatrelvir/ritonavir (Paxlovid) which is also based on anti-protease activity [32, 33].

Since the use of camostat mesylate did not affect the features of disease progression (infection’s clinical evolution, viral load kinetics, ICU admission, and mechanical ventilation) reduction in mortality rates is unlikely to be achieved. Additionally, the absence of a significant decrease in hospitalization rates signifies that camostat mesylate has low benefits in patients at risk for severe COVID-19. Furthermore, the no change in time for clinical recovery among camostat-treated groups indicates that this drug may be a non-useful strategy to treat COVID-19 outpatients with both moderate and mild forms.

Moreover, the earlier control of viral replication is essential for an antiviral drug to be effective in COVID-19 patients [34]. On one hand, this would prevent the tissular injury induced by either SARS-CoV-2 or its associated inflammation, and on the other hand, it would decrease the infectivity of patients, thereby minimizing disease transmission. The anti-SARS-CoV-2 activity of camostat mesylate was speculated from its potential to block TMPRSS2-mediated viral fusion; thus, inhibiting viral replication in host cells, as shown by in vitro human cell and animal studies [35, 36]. The fact that camostat mesylate did not induce significant acceleration in PCR negativation time likely reflects its failure to effectively contribute to viral clearance and replication arrest/prevention.

Mechanistically, this seems to be due to two main reasons: (i) the non-pharmacological effectiveness of camostat mesylate as a TMPRSS2 inhibitor administered in monotherapy, or (ii) the non-utility of TMPRSS2 inhibition as an exclusive strategy to prevent viral invasion (the most likely probability). Hence, studies on the molecular pharmacology of camostat mesylate indicated that it may not be the optimal ligand to block TMPRSS2 activity [37–39]. Notably, it has been revealed that camostat has lesser inhibition potential compared to a similar TMPRSS2 blocker nafamostat as the latter forms significantly higher amounts of enzyme-substrate stable complexes [39]. Remarkably, the pharmacological potency of camostat mesylate was shown to be 10-fold less than that of nafamostat mesylate [2]. Further results from animal studies concluded that nafamostat is a better candidate for the prevention of SARS-CoV-2 TMPRSS2-mediated entry compared to camostat [40]. Simultaneously, it has been recently demonstrated that SARS-CoV-2 can enter target cells without the need for ACE2 and TMPRSS2 participation through “cell-to-cell fusion” mechanism. Notably, the involvement of TMPRSS2 in this mechanism was found to be dispensable suggesting that SARS-CoV-2 exhibits TMPRSS2-independent cellular invasion strategies [41].

Moreover, even in the absence of TMPRSS2, SARS-CoV-2 has an alternative route of entry by endocytosis and transportation into endolysosomes where it is released to the cytosol via the action of acid-activated cathepsin L protease [42]. Therefore, SARS-CoV-2 can use these pathways to escape from camostat mesylate and other specific inhibitors of TMPRSS2. This possibility is more pronounced with the novel SARS-CoV-2 variants (i.e., Omicron) which no longer rely on TMPRSS-2 as a fusogenicity factor [43]. Consequently, targeting TMPRSS2 alone is not sufficient to fully prevent penetration of SARS-CoV-2 to host cells. Another potential disadvantage of targeting TMPRSS2 is that this protein displays an interindividual structural variability with some functional variants being expressed at relatively high frequencies among many human populations [44]. There are also interindividual quantitative variations in TMPRSS2 levels secondary to genetic polymorphisms across populations [45]. Both qualitative and quantitative variations in TMPRSS2 may alter the individuals’ response to camostat mesylate and similar drugs by potentially decreasing ligand potency and efficiency.

Besides the low efficacy profile, analysis of the safety profile indicated some concerns with camostat mesylate due to a higher risk of any serious adverse events in the treated groups compared to controls. The mechanisms of this molecule’s toxicity are unclear; however, since TMPRSS2 is ubiquitously expressed in the human body its inhibition may result in systemic undesirable effects. Additionally, camostat mesylate has a broad action on other proteases involved in multiple functions such as blood pressure control and renal function, inflammation, and coagulation [46]; which when inhibited in COVID-19 patients (especially those with severe forms) may lead to more harms than goods. Worth mentioning that camostat mesylate has anti-diarrheic effects as it was shown to normalize intestinal hyperpermeability in rats which could explain the lower susceptibility to diarrhea in COVID-19 patients compared to placebo [43].

### Strengths and limitations

To the best of our knowledge, this is the first meta-analysis that assesses the safety and efficacy of camostat mesylate in COVID-19 patients. Therefore, this paper presents the gold-standard evidence on this topic including all available RCTs that met our criteria to reach the highest accessible quality of evidence. We analyzed data from a large number (*n* = 1,623) of patients and provided key findings. However our paper is undermined by the following: first, we included three non-peer-reviewed reports, including a preprint ref and two unpublished RCTs data [27, 30]. Second, the included studies suffered from significant heterogeneity in the camostat dosing regimen, which can affect our findings. Third, all the included studies recruited patients with mild to moderate COVID-19, with only Gunst et al. and Karolyi et al. [26, 29], recruiting hospitalized patients with moderate to severe disease; therefore, our results may not be generalizable for severe COVID-19.

### Implications and future perspectives

Targeting viral entry is a well-established strategy to fight viral diseases such as HIV and influenza virus infections; however, its benefit in COVID-19 remains questionable and is not yet supported by robust quality of evidence. Until full data becomes available, the results in this study do not exclude the usefulness of camostat mesylate in the context of COVID-19 infection as co-administration with other synergistic antiviral drugs may boost its efficacy profile. Since furin, another transmembranous enzyme involved in the proteolytic processing of SARS-CoV-2 is necessary for TMPRSS2-independent fusion (i.e., cell-to-cell fusion), the combination of furin and TMPRSS2 inhibitors may enhance the overall preventive effects on viral entry and infectivity [13, 38]. Nevertheless, the constant changes in SARS-CoV-2 cellular invasion pathways may not facilitate the development of the most adequate combination for viral entry inhibitors. Importantly, the presence of safety concerns with camostat mesylate use among COVID-19 patients should justify more caution and strict patient monitoring in future evaluations. Based on these concerns and the lack of proof of effectiveness, current guidelines should recommend against the use of camostat mesylate in COVID-19 patients outside the context of clinical trials.

## Conclusion

The current evidence does not support the efficacy of camostat mesylate in treating COVID-19 infection. Rather, it indicates some safety concerns that should be considered before further testing this drug in large-scale trials. Nevertheless, since the available data is incomplete more RCTs are still required to conclude the therapeutic benefit of camostat mesylate in COVID-19. At the same time, it might also be worthy to continue investigating the utility of viral entry inhibitors as potential treatment for COVID-19 by focusing on other TMPRSS2 inhibitors with greater pharmacological potency, agents with TMPRSS2-independent activity, or effective synergistic combinations of both.

### Electronic supplementary material

Below is the link to the electronic supplementary material.

Supplementary Material 1

## Data Availability

All data generated or analysed during this study are included in this published article [and its supplementary information files].
