# Supplementary material for: Safety and Efficacy of Camostat Mesylate for Covid-19: a systematic review and Meta-analysis of Randomized controlled trials
Source: BMC Infect Dis. 2024 Jul 19;24:709. doi: 10.1186/s12879-024-09468-w (PMC11264738; doi:10.1186/s12879-024-09468-w)
Supplement: Supplementary file 1 — Supplementary Material 1 [file 12879_2024_9468_MOESM1_ESM.docx]

**Title.**

**Safety and Efficacy of Camostat Mesylate for Covid-19: A Systematic Review and Meta-Analysis of Randomized Controlled Trials.**

**Running Title**

Camostat Mesylate for Covid-19.

**Authors.**

Ubaid Khan ^1^, Muhammad Mubariz ^2^, Yahia Khlidj^3^, Muhammad Moiz Nasir ^4^, Shrouk Ramadan^5^, Fatima Saeed^1^, Aiman Muhammad^6^, Mohamed Abuelazm^7^.

**Affiliations.**

1. King Edward Medical University, Lahore, Pakistan.
2. Akhtar Saeed Medical and Dental College, Lahore, Pakistan.
3. Dow University of health science, Karachi, Pakistan.
4. Faculty of medicine, Algiers University.
5. Faculty of medicine Ain Shams University, Cairo, Egypt.
6. Khyber Girls Medical College, Peshawar, Pakistan.
7. Faculty of Medicine, Tanta University, Tanta, Egypt

**Keywords.**

Camostat Mesylate, Covid-19, Pandemic, SARS-CoV-2, review, analysis.

**Tables.**

**Table S1:** Data source and search strategy.

**Table S2:** Reason for exclusion of studies in full text screening.

**Table S3:** Detailed explanation of serious adverse events definition.

**Table S4-S12:** Detailed risk of bias assessment for each trial.

**Table S13:** Sensitivity analysis.

.

| Database | Search Terms/ | Search Field | Search Results |
| --- | --- | --- | --- |
| Pubmed | (camostate OR "camostat mesylate" OR "camostat mesilate" OR methanesulfonate OR camostate-mesilate OR "FOY 305" OR FOY-305 OR "FOY S 980" OR Foipan OR Foypan) AND (Coronavirus OR “Coronavirus infections” OR “COVID 2019” OR SARS2 OR SARS-CoV-2 OR “SARS-CoV-19” OR “novel coronavirus disease” OR “coronavirus infection” OR “novel CoV” OR “2019 ncov” OR “sars cov2” OR cov2 OR ncov OR COVID-19 OR COVID19 OR coronaviridae OR coronavirus) | All Field | 274 |
| Cochrane | (camostate OR "camostat mesylate" OR "camostat mesilate" OR methanesulfonate OR camostate-mesilate OR "FOY 305" OR FOY-305 OR "FOY S 980" OR Foipan OR Foypan) AND (Coronavirus OR “Coronavirus infections” OR “COVID 2019” OR SARS2 OR SARS-CoV-2 OR “SARS-CoV-19” OR “novel coronavirus disease” OR “coronavirus infection” OR “novel CoV” OR “2019 ncov” OR “sars cov2” OR cov2 OR ncov OR COVID-19 OR COVID19 OR coronaviridae OR coronavirus) | All Field | 33 |
| WOS | (camostate OR "camostat mesylate" OR "camostat mesilate" OR methanesulfonate OR camostate-mesilate OR "FOY 305" OR FOY-305 OR "FOY S 980" OR Foipan OR Foypan) AND (Coronavirus OR “Coronavirus infections” OR “COVID 2019” OR SARS2 OR SARS-CoV-2 OR “SARS-CoV-19” OR “novel coronavirus disease” OR “coronavirus infection” OR “novel CoV” OR “2019 ncov” OR “sars cov2” OR cov2 OR ncov OR COVID-19 OR COVID19 OR coronaviridae OR coronavirus) | All Field | 67 |
| SCOPUS | (camostate OR "camostat mesylate" OR "camostat mesilate" OR methanesulfonate OR camostate-mesilate OR "FOY 305" OR FOY-305 OR "FOY S 980" OR Foipan OR Foypan) AND (Coronavirus OR “Coronavirus infections” OR “COVID 2019” OR SARS2 OR SARS-CoV-2 OR “SARS-CoV-19” OR “novel coronavirus disease” OR “coronavirus infection” OR “novel CoV” OR “2019 ncov” OR “sars cov2” OR cov2 OR ncov OR COVID-19 OR COVID19 OR coronaviridae OR coronavirus) | all | 379 |
| Clinical trails | (camostate OR "camostat mesylate" OR "camostat mesilate" OR methanesulfonate OR camostate-mesilate OR "FOY 305" OR FOY-305 OR "FOY S 980" OR Foipan OR Foypan) AND (Coronavirus OR “Coronavirus infections” OR “COVID 2019) | All Field | 41 |
| medrxiv | (camostate OR "camostat mesylate" OR "camostat mesilate" OR methanesulfonate OR camostate-mesilate OR "FOY 305" OR FOY-305 OR "FOY S 980" OR Foipan OR Foypan) AND (Coronavirus OR “Coronavirus infections” OR “COVID 2019) | All field | 22 |

Table S1 Data source and search strategy

| Title | Authors | Published Year | Accession Number | DOI | Covidence # | Study | Notes |
| --- | --- | --- | --- | --- | --- | --- | --- |
| Reconvalescent Plasma/Camostat Mesylate Early in SARS-CoV-2 Q-PCR (COVID-19) Positive High-risk Individuals | NCT04681430, | 2020 | CN-02209590 | NA | #1 | NCT04681430 2020 | Exclusion reason: duplicated; |
| Effect of Camostat Mesilate on individuals with COVID-19 infection | EUCTR2020-001200-42-DK, | 2020 | CN-02169617 | NA | #2 | EUCTR2020-001200-42-DK 2020 | Exclusion reason: Incomplete study; |
| Effect of Camostate mesylate on the outcome of Coronavirus (COVID-19)-induced pneumonia | IRCT20200317046797N1, | 2020 | CN-02171573 | NA | #3 | IRCT20200317046797N1 2020 | Exclusion reason: Incomplete study; |
| A clinical study in a community setting to see whether use of camostat reduces the worsening of COVID-19 | EUCTR2020-002110-41-GB, | 2020 | CN-02169809 | NA | #4 | EUCTR2020-002110-41-GB 2020 | Exclusion reason: Incomplete study; |
| Camostat and Artemisia Annua vs Placebo in COVID-19 Outpatients | NCT04530617, | 2020 | CN-02163219 | NA | #6 | NCT04530617 2020 | Exclusion reason: Incomplete study; |
| FOY-305-03: FOY-305 TQT study(COVID-19) | jRCT2071200109, | 2021 | CN-02256917 | NA | #7 | jRCT2071200109 2021 | Exclusion reason: Incomplete study; |
| CAMOVID: a multicenter randomized trial to evaluate the efficacy and safety of camostat mesylate for the treatment of SARS-CoV-2 infection in ambulatory adult patients | EUCTR2020-003366-39-FR, | 2020 | CN-02187053 | NA | #11 | EUCTR2020-003366-39-FR 2020 | Exclusion reason: Incomplete study; |
| Camostat Mesilate Treating Patients With Hospitalized Patients With COVID-19 | NCT04470544, | 2020 | CN-02130126 | NA | #17 | NCT04470544 2020 | Exclusion reason: Incomplete study; |
| A multicenter, double-blind, randomized, parallel-group, placebo-controlled study to evaluate the efficacy and safety of camostat mesilate in patients with COVID-19 (CANDLE study) | Kinoshita, T; Shinoda, M; Nishizaki, Y; Shiraki, K; Hirai, Y; Kichikawa, Y; Tsushima, K; Shinkai, M; Komura, N; Yoshida, K; et al. | 2022 | CN-02466027 | NA | #20 | Kinoshita 2022 | Exclusion reason: duplicated; |
| Plasma from Covid 19 recovered patients or camostat as a therapy for early SARS-CoV-2 infection in high risk individuals | EUCTR2020-004695-18-DE, | 2020 | CN-02241381 | NA | #22 | EUCTR2020-004695-18-DE 2020 | Exclusion reason: Incomplete study; |
| CAMOVID : evaluation of Efficacy and Safety of Camostat Mesylate for the Treatment of SARS-CoV-2 Infection - COVID-19 in Ambulatory Adult Patients | NCT04608266, | 2020 | CN-02197018 | NA | #26 | NCT04608266 2020 | Exclusion reason: Study terminated; |
| Correction: a multicenter, double-blind, randomized, parallel-group, placebo-controlled study to evaluate the efficacy and safety of camostat mesilate in patients with COVID-19 (CANDLE study) (BMC Medicine, (2022), 20, 1, (342), 10.1186/s12916-022-02518- | Kinoshita, T; Shinoda, M; Nishizaki, Y; Shiraki, K; Hirai, Y; Kichikawa, Y; Tsushima, K; Shinkai, M; Komura, N; Yoshida, K; et al. | 2022 | CN-02514946 | NA | #31 | Kinoshita 2022 | Exclusion reason: duplicated; |
| The Utility of Camostat Mesylate in Patients With COVID-19 Associated Coagulopathy (CAC) and Cardiovascular Complications | NCT04435015, | 2020 | CN-02133672 | NA | #32 | NCT04435015 2020 | Exclusion reason: duplicated; |
| Camostat Mesilate contained oral wash gargle trial | jRCTs031200113, | 2020 | CN-02188563 | NA | #33 | jRCTs031200113 2020 | Exclusion reason: Wrong intervention; |
| Camostat mesylate inhibits SARS-CoV-2 activation by TMPRSS2-related proteases and its metabolite GBPA exerts antiviral activity | Hoffmann, M; Hofmann-Winkler, H; Smith, JC; Kruger, N; Arora, P; Sorensen, LK; Sogaard, OS; Hasselstrom, JB; Winkler, M; Hempel, T; Raich, L; Olsson, S; Danov, O; Jonigk, D; Yamazoe, T; Yamatsuta, K; Mizuno, H; Ludwig, S; Noe, F; Kjolby, M; Braun, A; Sheltzer, JM; Pohlmann, S | 2021 | WOS:000634285400009 | 10.1016/j.ebiom.2021.103255 | #41 | Hoffmann 2021 | Exclusion reason: Wrong study design; |
| Inhibition of SARS-CoV-2 entry through the ACE2/TMPRSS2 pathway: a promising approach for uncovering early COVID-19 drug therapies | Ragia, G; Manolopoulos, VG | 2020 | WOS:000551052800001 | 10.1007/s00228-020-02963-4 | #47 | Ragia 2020 | Exclusion reason: Wrong study design; |
| Virtual Screening of Natural Products against Type II Transmembrane Serine Protease (TMPRSS2), the Priming Agent of Coronavirus 2 (SARS-CoV-2) | Rahman, N; Basharat, Z; Yousuf, M; Castaldo, G; Rastrelli, L; Khan, H | 2020 | WOS:000539293400004 | 10.3390/molecules25102271 | #49 | Rahman 2020 | Exclusion reason: Wrong intervention; |
| Virtual drug repurposing study against SARS-CoV-2 TMPRSS2 target | Durdagi, S | 2020 | WOS:000541522100008 | 10.3906/biy-2005-112 | #50 | Durdagi 2020 | Exclusion reason: Wrong intervention; |
| Low risk of the TMPRSS2 inhibitor camostat mesylate and its metabolite GBPA to act as perpetrators of drug-drug interactions | Weiss, J; Bajraktari-Sylejmani, G; Haefeli, WE | 2021 | WOS:000628895400011 | 10.1016/j.cbi.2021.109428 | #53 | Weiss 2021 | Exclusion reason: Wrong study design; |
| Withanone and Withaferin-A are predicted to interact with transmembrane protease serine 2 (TMPRSS2) and block entry of SARS-CoV-2 into cells | Kumar, V; Dhanjal, JK; Bhargava, P; Kaul, A; Wang, J; Zhang, H; Kaul, SC; Wadhwa, R; Sundar, D | 2022 | WOS:000544520800001 | 10.1080/07391102.2020.1775704 | #62 | Kumar 2022 | Exclusion reason: Wrong study design; |
| A common TMPRSS2 variant has a protective effect against severe COVID-19 | GenOMICC Consortium; ISARIC4C Investigators; David, A; Parkinson, N; Peacock, TP; Pairo-Castineira, E; Khanna, T; Cobat, A; Tenesa, A; Sancho-Shimizu, V; Casanova, JL; Abel, L; Barclay, WS; Baillie, JK; Sternberg, MJE | 2022 | WOS:000820180500007 | 10.1016/j.retram.2022.103333 | #65 | GenOMICCConsortium 2022 | Exclusion reason: Wrong study design; |
| A computational study of potential therapeutics for COVID-19 invoking conceptual density functional theory | Saloni; Kumari, D; Ranjan, P; Chakraborty, T | 2022 | WOS:000852272500001 | 10.1007/s11224-022-02048-1 | #68 | Saloni 2022 | Exclusion reason: Wrong study design; |
| Peptidomimetic inhibitors of TMPRSS2 block SARS-CoV-2 infection in cell culture | Wettstein, L; Knaff, PM; Kersten, C; Muller, P; Weil, T; Conzelmann, C; Muller, JA; Bruckner, M; Hoffmann, M; Pohlmann, S; Schirmeister, T; Landfester, K; Munch, J; Mailander, V | 2022 | WOS:000823718100001 | 10.1038/s42003-022-03613-4 | #71 | Wettstein 2022 | Exclusion reason: Wrong study design; |
| Exploring the anti-SARS-CoV-2 main protease potential of FDA approved marine drugs using integrated machine learning templates as predictive tools | Attiq, N; Arshad, U; Brogi, S; Shafiq, N; Imtiaz, F; Parveen, S; Rashid, M; Noor, N | 2022 | WOS:000861503900001 | 10.1016/j.ijbiomac.2022.09.086 | #72 | Attiq 2022 | Exclusion reason: Wrong study design; |
| The discovery and development of transmembrane serine protease 2 (TMPRSS2) inhibitors as candidate drugs for the treatment of COVID-19 | Mantzourani, C; Vasilakaki, S; Gerogianni, VE; Kokotos, G | 2022 | WOS:000746421600001 | 10.1080/17460441.2022.2029843 | #73 | Mantzourani 2022 | Exclusion reason: Wrong study design; |
| Camostat mesilate therapy for COVID-19 | Uno, Y | 2020 | WOS:000529459600001 | 10.1007/s11739-020-02345-9 | #77 | Uno 2020 | Exclusion reason: Wrong study design; |
| Camostat mesylate therapy in critically ill patients with COVID-19 pneumonia | UAE-Jena Res Grp; Sakr, Y; Bensasi, H; Taha, A; Bauer, M; Ismail, K | 2021 | WOS:000639380700002 | 10.1007/s00134-021-06395-1 | #93 | UAE-JenaResGrp 2021 | Exclusion reason: Wrong comparator; |
| A multicenter, double-blind, randomized, parallel-group, placebo-controlled study to evaluate the efficacy and safety of camostat mesilate in patients with COVID-19 (CANDLE study) (vol 20, 342, 2022) | Kinoshita, T; Shinoda, M; Nishizaki, Y; Shiraki, K; Hirai, Y; Kichikawa, Y; Tsushima, K; Shinkai, M; Komura, N; Yoshida, K; Kido, Y; Kakeya, H; Uemura, N; Kadota, J | 2022 | WOS:000895935400002 | 10.1186/s12916-022-02695-5 | #96 | Kinoshita 2022 | Exclusion reason: duplicated; |
| A PHASE I STUDY TO EVALUATE SAFETY, TOLERABILITY AND PHARMACOKINETICS OF HIGH DOSES OF CAMOSTAT MESYLATE IN HEALTHY SUBJECTS PROVIDES A RATIONALE TO REPURPOSE THE TMPRSS2 INHIBITOR FOR THE TREATMENT OF COVID-19. | Kitagawa, J; Arai, H; Iida, H; Mukai, J; Furukawa, K; Ohtsu, S; Nakade, S; Hikima, T; Haranaka, M; Uemura, N | 2021 | WOS:000619875100129 |  | #97 | Kitagawa 2021 | Exclusion reason: Wrong patient population; |
| Correction: A multicenter, double-blind, randomized, parallel-group, placebo-controlled study to evaluate the efficacy and safety of camostat mesilate in patients with COVID-19 (CANDLE study) (BMC Medicine, (2022), 20, 1, (342), 10.1186/s12916-022-02518-7 | Kinoshita, T.; Shinoda, M.; Nishizaki, Y.; Shiraki, K.; Hirai, Y.; Kichikawa, Y.; Tsushima, K.; Shinkai, M.; Komura, N.; Yoshida, K.; Kido, Y.; Kakeya, H.; Uemura, N.; Kadota, J. | 2022 | NA | 10.1186/s12916-022-02695-5 | #105 | Kinoshita 2022 | Exclusion reason: duplicated; |
| Spiking dependence of SARS-CoV-2 pathogenicity on TMPRSS2 | Abbasi, A.Z.; Kiyani, D.A.; Hamid, S.M.; Saalim, M.; Fahim, A.; Jalal, N. | 2021 | NA | 10.1002/jmv.26911 | #252 | Abbasi 2021 | Exclusion reason: duplicated; |
| Computational screening of phytochemicals from three medicinal plants as inhibitors of transmembrane protease serine 2 implicated in SARS-CoV-2 infection | Oyedara, O.O.; Agbedahunsi, J.M.; Adeyemi, F.M.; JuÃ¡rez-Saldivar, A.; Fadare, O.A.; Adetunji, C.O.; Rivera, G. | 2021 | NA | 10.1016/j.phyplu.2021.100135 | #283 | Oyedara 2021 | Exclusion reason: Wrong intervention; |
| Targeting the entry step of SARS-CoV-2: a promising therapeutic approach | Li, J.; Zhan, P.; Liu, X. | 2020 | NA | 10.1038/s41392-020-0195-x | #339 | Li 2020 | Exclusion reason: Wrong study design; |
| From Japan to Denmark: Camostat mesilate for COVID-19 | Siebenand, S. | 2020 | NA |  | #446 | Siebenand 2020 | Exclusion reason: Wrong study design; |
| A novel class of TMPRSS2 inhibitors potently block SARS-CoV-2 and MERS-CoV viral entry and protect human epithelial lung cells. | Mahoney M; Damalanka VC; Tartell MA; Chung DH; LourenÃ§o AL; Pwee D; Mayer Bridwell AE; Hoffmann M; Voss J; Karmakar P; Azouz NP; Klingler AM; Rothlauf PW; Thompson CE; Lee M; Klampfer L; Stallings CL; Rothenberg ME; PÃ¶hlmann S; Whelan SPJ; O'Donoghue AJ; Craik CS; Janetka JW | 2021 | NA | 10.1073/pnas.2108728118 | #499 | Mahoney 2021 | Exclusion reason: Wrong study design; |
| Camostat mesylate inhibits SARS-CoV-2 activation by TMPRSS2-related proteases and its metabolite GBPA exerts antiviral activity. | Hoffmann M; Hofmann-Winkler H; Smith JC; KrÃ¼ger N; SÃ¸rensen LK; SÃ¸gaard OS; HasselstrÃ¸m JB; Winkler M; Hempel T; Raich L; Olsson S; Yamazoe T; Yamatsuta K; Mizuno H; Ludwig S; NoÃ© F; Sheltzer JM; Kjolby M; PÃ¶hlmann S | 2020 | NA | 10.1101/2020.08.05.237651 | #522 | Hoffmann 2020 | Exclusion reason: Wrong outcomes; |
| Evaluation of intranasal nafamostat or camostat for SARS-CoV-2 chemoprophylaxis in Syrian golden hamsters. | Neary M; Box H; Sharp J; Tatham L; Curley P; Herriott J; Kijak E; Arshad U; Hobson JJ; Rajoli R; Pertinez H; Valentijn A; Dhaliwal K; McCaughan F; Rannard SP; Kipar A; Stewart JP; Owen A | 2021 | NA | 10.1101/2021.07.08.451654 | #530 | Neary 2021 | Exclusion reason: Wrong patient population; |
| Spontaneous binding of potential COVID-19 drugs (Camostat and Nafamostat) to human serine protease TMPRSS2. | Zhu H; Du W; Song M; Liu Q; Herrmann A; Huang Q | 2021 | NA | 10.1016/j.csbj.2020.12.035 | #531 | Zhu 2021 | Exclusion reason: Wrong study design; |
| Computational screening of camostat and related compounds against human TMPRSS2: A potential treatment of COVID-19. | Sharma T; Baig MH; Khan MI; Alotaibi SS; Alorabi M; Dong JJ | 2022 | NA | 10.1016/j.jsps.2022.01.005 | #548 | Sharma 2022 | Exclusion reason: Wrong study design; |
| Favipiravir, camostat, and ciclesonide combination therapy in patients with moderate COVID-19 pneumonia with/without oxygen therapy: An open-label, single-center phase 3 randomized clinical trial. | Terada J; Fujita R; Kawahara T; Hirasawa Y; Kinoshita T; Takeshita Y; Isaka Y; Kinouchi T; Tajima H; Tada Y; Tsushima K | 2022 | NA | 10.1016/j.eclinm.2022.101484 | #565 | Terada 2022 | Exclusion reason: Wrong intervention; |
| In silico study on spice-derived antiviral phytochemicals against SARS-CoV-2 TMPRSS2 target. | Yadav PK; Jaiswal A; Singh RK | 2022 | NA | 10.1080/07391102.2021.1965658 | #602 | Yadav 2022 | Exclusion reason: Wrong intervention; |
| SPIKE-1: A Randomised Phase II/III trial in a community setting, assessing use of camostat in reducing the clinical progression of COVID-19 by blocking SARS-CoV-2 Spike protein-initiated membrane fusion. | Halford S; Wan S; Dragoni I; Silvester J; Nazarov B; Anthony D; Anthony S; Ladds E; Norrie J; Dhaliwal K | 2021 | ClinicalTrials.gov/NCT04455815 | 10.1186/s13063-021-05461-9 | #653 | Halford 2021 | Exclusion reason: Incomplete study; |
| SARS-CoV-2 Infection of Human Neurons Is TMPRSS2 Independent, Requires Endosomal Cell Entry, and Can Be Blocked by Inhibitors of Host Phosphoinositol-5 Kinase. | Kettunen P; Lesnikova A; RÃ¤sÃ¤nen N; Ojha R; Palmunen L; Laakso M; Lehtonen Å ; Kuusisto J; PietilÃ¤inen O; Saber SH; Joensuu M; Vapalahti OP; Koistinaho J; Rolova T; Balistreri G | 2023 | NA | 10.1128/jvi.00144-23 | #659 | Kettunen 2023 | Exclusion reason: Wrong study design; |
| Screening strategy of TMPRSS2 inhibitors by FRET-based enzymatic activity for TMPRSS2-based cancer and COVID-19 treatment. | Chen Y; Huang WC; Yang CS; Cheng FJ; Chiu YF; Chen HF; Huynh TK; Huang CF; Chen CH; Wang HC; Hung MC | 2021 | NA | NA | #667 | Chen 2021 | Exclusion reason: Wrong study design; |
| An Enzymatic TMPRSS2 Assay for Assessment of Clinical Candidates and Discovery of Inhibitors as Potential Treatment of COVID-19. | Shrimp JH; Kales SC; Sanderson PE; Simeonov A; Shen M; Hall MD | 2020 | NA | 10.1101/2020.06.23.167544 | #676 | Shrimp 2020 | Exclusion reason: Wrong study design; |
| Homology Modeling of TMPRSS2 Yields Candidate Drugs That May Inhibit Entry of SARS-CoV-2 into Human Cells. | Rensi S; Altman RB; Liu T; Lo YC; McInnes G; Derry A; Keys A | 2020 | NA | 10.26434/chemrxiv.12009582.v1 10.26434/chemrxiv.12009582 | #684 | Rensi 2020 | Exclusion reason: Wrong study design; |
| A Suite of TMPRSS2 Assays for Screening Drug Repurposing Candidates as Potential Treatments of COVID-19. | Shrimp JH; Janiszewski J; Chen CZ; Xu M; Wilson KM; Kales SC; Sanderson PE; Shinn P; Itkin Z; Guo H; Shen M; Klumpp-Thomas C; Michael SG; Zheng W; Simeonov A; Hall MD | 2022 | NA | 10.1101/2022.02.04.479134 | #688 | Shrimp 2022 | Exclusion reason: Wrong intervention; |
| Randomised controlled trial of intravenous nafamostat mesylate in COVID pneumonitis: Phase 1b/2a experimental study to investigate safety, Pharmacokinetics and Pharmacodynamics. | Quinn TM; Gaughan EE; Bruce A; Antonelli J; O'Connor R; Li F; McNamara S; Koch O; MacKintosh C; Dockrell D; Walsh T; Blyth KG; Church C; Schwarze J; Boz C; Valanciute A; Burgess M; Emanuel P; Mills B; Rinaldi G; Hardisty G; Mills R; Findlay EG; Jabbal S; Duncan A; Plant S; Marshall ADL; Young I; Russell K; Scholefield E; Nimmo AF; Nazarov IB; Churchill GC; McCullagh JSO; Ebrahimi KH; Ferrett C; Templeton K; Rannard S; Owen A; Moore A; Finlayson K; Shankar-Hari M; Norrie J; Parker RA; Akram AR; Anthony DC; Dear JW; Hirani N; Dhaliwal K | 2022 | NA | 10.1016/j.ebiom.2022.103856 | #700 | Quinn 2022 | Exclusion reason: Wrong intervention; |
| Correction to: SPIKE-1: A Randomised Phase II/III trial in a community setting, assessing use of camostat in reducing the clinical progression of COVID-19 by blocking SARS-CoV-2 Spike protein-initiated membrane fusion. | Halford S; Wan S; Dragoni I; Silvester J; Nazarov B; Anthony D; Anthony S; Ladds E; Norrie J; Dhaliwal K | 2022 | NA | 10.1186/s13063-022-06266-0 | #724 | Halford 2022 | Exclusion reason: duplicated; |
| Middle East respiratory syndrome coronavirus infection mediated by the transmembrane serine protease TMPRSS2. | Shirato K; Kawase M; Matsuyama S | 2013 | NA | 10.1128/JVI.01890-13 | #730 | Shirato 2013 | Exclusion reason: Wrong intervention; |
| Identification of potential anti-TMPRSS2 natural products through homology modelling, virtual screening and molecular dynamics simulation studies. | Chikhale RV; Gupta VK; Eldesoky GE; Wabaidur SM; Patil SA; Islam MA | 2020 | NA | 10.1080/07391102.2020.1798813 | #733 | Chikhale 2020 | Exclusion reason: Wrong study design; |
| FoistarÂ®(Camostat mesylate) associated with the significant decrease in CRP levels compared to KaletraÂ®(Lopinavir/Ritonavir) treatment in Korean mild COVID-19 pneumonic patients | Jae-Phil Choi; Hyoung-Jun Kim; Jumi Han; SuJung Park; JinJoo Han | 2020 | NA | 10.1101/2020.12.10.20240689 | #755 | Jae-PhilChoi 2020 | Exclusion reason: Wrong comparator; |
| The Impact of Camostat Mesilate on COVID-19 Infection | University of Aarhus | 2020 | NCT04321096 | NA | #776 | UniversityofAarhus 2020 | Exclusion reason: Incomplete study; |
| Camostat Mesilate Treating Patients With Hospitalized Patients With COVID-19 | Alan Bryce; Academic; Community Cancer Research United | 2020 | NCT04470544 | NA | #780 | AlanBryce 2020 | Exclusion reason: Incomplete study; |
| Reconvalescent Plasma/Camostat Mesylate Early in SARS-CoV-2 Q-PCR (COVID-19) Positive High-risk Individuals | Heinrich-Heine University, Duesseldorf; The Federal Ministry of Health, Germany | 2021 | NCT04681430 | NA | #790 | Heinrich-HeineUniversity 2021 | Exclusion reason: Wrong intervention; |
| Camostat With Bicalutamide for COVID-19 | Sidney Kimmel Comprehensive Cancer Center at Johns Hopkins | 2021 | NCT04652765 | NA | #791 | SidneyKimmelComprehensiveCancerCenteratJohnsHopkins 2021 | Exclusion reason: Study terminated; |
| Clinical Efficacy of Nafamostat Mesylate for COVID-19 Pneumonia | Gyeongsang National University Hospital | 2020 | NCT04418128 | NA | #792 | GyeongsangNationalUniversityHospital 2020 | Exclusion reason: Incomplete study; |
| CAMOVID : Evaluation of Efficacy and Safety of Camostat Mesylate for the Treatment of SARS-CoV-2 Infection - COVID-19 in Ambulatory Adult Patients | Assistance Publique - HÃ´pitaux de Paris | 2020 | NCT04608266 | NA | #803 | AssistancePublique-HÃ´pitauxdeParis 2020 | Exclusion reason: Study terminated; |
| A Trial Looking at the Use of Camostat in People Who Have Tested Positive for Coronavirus (COVID-19) (SPIKE-1) | Cancer Research UK; Latus Therapeutics | 2020 | NCT04455815 | NA | #807 | CancerResearch 2020 | Exclusion reason: duplicated; |
| COVID-19 Outpatient Pragmatic Platform Study (COPPS) - Camostat Sub-Protocol | Stanford University | 2021 | NCT04662073 | NA | #811 | StanfordUniversity 2021 | Exclusion reason: Wrong study design; |
| The Utility of Camostat Mesylate in Patients With COVID-19 Associated Coagulopathy (CAC) and Cardiovascular Complications | Yale University; Ono Pharmaceutical Co. Ltd | 2021 | NCT04435015 | NA | #814 | YaleUniversity 2021 | Exclusion reason: Study terminated; |

Table S2, Reason for exclusion of studies in full text screening.

| **Study ID** | | **Definition of Serious Adverse Events** |
| --- | --- | --- |
|  |  |  |
| 1 | **Chupp et al. 2022** | No specific definition was reported |
| 2 | **Karolyi et al. 2022** | No serious adverse event was reported . |
| 3 | **Jilg et al. 2023** | Development of grade 3 or higher through 28 days |
| 4 | **Kim et al. 2022** | No serious adverse drug reactions were reported |
| 5 | **Tobback et al. 2022** | **No specific definition** was reported |
| 6 | **Gunst et al. 2021** | Was characterized as any adverse medical event that, regardless of dosage, led to death, posed a threat to life, necessitated hospitalization either as an inpatient or extended existing hospital stays or held medical significance. |
| 7 | **Kinoshita et al. 2022** | No serious adverse event related to the study was reported. |
| 8 | **NCT04524663** | N/A |
| 9 | **NCT04583592** | N/A |

***Table S3:*** Detailed explanation of serious adverse events definition.

| **Domain** | **Signalling question** | **Response** | **Comments** |
| --- | --- | --- | --- |
| **Bias arising from the randomization process** | 1.1 Was the allocation sequence random? | Y | Participants were randomized by a computer generated randomization process in a 1:1 ratio. The allocation sequence was concealed. |
|  | 1.2 Was the allocation sequence concealed until participants were enrolled and assigned to interventions? | Y |  |
|  | 1.3 Did baseline differences between intervention groups suggest a problem with the randomization process? | N | No baseline differences |
|  | **Risk of bias judgement** | **Low** |  |
| **Bias due to deviations from intended interventions** | 2.1.Were participants aware of their assigned intervention during the trial? | N | Subjects, caregivers, investigational site personnel, study sponsor study team members, and all other study personnel remained blinded to the identity of the treatment assignments; assignments were be available only to the unblinded randomization team, the DSMB for the study (if requested), and drug safety personnel who were not part of the study team |
|  | 2.2.Were carers and people delivering the interventions aware of participants' assigned intervention during the trial? | N |  |
|  | 2.3. If Y/PY/NI to 2.1 or 2.2: Were there deviations from the intended intervention that arose because of the experimental context? | NA |  |
|  | 2.4 If Y/PY to 2.3: Were these deviations likely to have affected the outcome? | NA |  |
|  | 2.5. If Y/PY/NI to 2.4: Were these deviations from intended intervention balanced between groups? | NA |  |
|  | 2.6 Was an appropriate analysis used to estimate the effect of assignment to intervention? | Y | ITT |
|  | 2.7 If N/PN/NI to 2.6: Was there potential for a substantial impact (on the result) of the failure to analyse participants in the group to which they were randomized? | NA |  |
|  | **Risk of bias judgement** | **Low** |  |
| **Bias due to missing outcome data** | 3.1 Were data for this outcome available for all, or nearly all, participants randomized? | Y | No missing outcomes |
|  | 3.2 If N/PN/NI to 3.1: Is there evidence that result was not biased by missing outcome data? | NA |  |
|  | 3.3 If N/PN to 3.2: Could missingness in the outcome depend on its true value? | NA |  |
|  | 3.4 If Y/PY/NI to 3.3: Is it likely that missingness in the outcome depended on its true value? | NA |  |
|  | **Risk of bias judgement** | **Low** |  |
| **Bias in measurement of the outcome** | 4.1 Was the method of measuring the outcome inappropriate? | N | The primary endpoint was change in the log10 viral load of a NP swab specimen as determined by quantitative RT-PCR testing from baseline to day 4 post-randomization |
|  | 4.2 Could measurement or ascertainment of the outcome have differed between intervention groups? | PN |  |
|  | 4.3 Were outcome assessors aware of the intervention received by study participants? | N | Outcome assessors were blinded to the treatment |
|  | 4.4 If Y/PY/NI to 4.3: Could assessment of the outcome have been influenced by knowledge of intervention received? | NA |  |
|  | 4.5 If Y/PY/NI to 4.4: Is it likely that assessment of the outcome was influenced by knowledge of intervention received? | NA |  |
|  | **Risk of bias judgement** | **Low** |  |
| **Bias in selection of the reported result** | 5.1 Were the data that produced this result analysed in accordance with a pre-specified analysis plan that was finalized before unblinded outcome data were available for analysis? | Y | The data analysis plan specified in the protocol is the same as the data that reported in the paper |
|  | 5.2 ... multiple eligible outcome measurements (e.g. scales, definitions, time points) within the outcome domain? | PN |  |
|  | 5.3 ... multiple eligible analyses of the data? | PN |  |
|  | **Risk of bias judgement** | **Low** |  |
| **Overall bias** | **Risk of bias judgement** | **Low** |  |

Table S4: Risk of bias assessment details of Chupp et al. 2022, using ROB2.

| **Domain** | **Signalling question** | **Response** | **Comments** |
| --- | --- | --- | --- |
| **Bias arising from the randomization process** | 1.1 Was the allocation sequence random? | Y | Each patient was randomized via an online tool which was provided by the medical university of Vienna and received a consecutive randomization number for the main study and a substudy if applicable.  Patients, nurses, doctors, and the study team were aware of the treatment allocation in this open-label trial. |
|  | 1.2 Was the allocation sequence concealed until participants were enrolled and assigned to interventions? | N |  |
|  | 1.3 Did baseline differences between intervention groups suggest a problem with the randomization process? | N | No baseline differences |
|  | **Risk of bias judgement** | **High** |  |
| **Bias due to deviations from intended interventions** | 2.1.Were participants aware of their assigned intervention during the trial? | Y | "Open-label" |
|  | 2.2.Were carers and people delivering the interventions aware of participants' assigned intervention during the trial? | Y |  |
|  | 2.3. If Y/PY/NI to 2.1 or 2.2: Were there deviations from the intended intervention that arose because of the experimental context? | PN | No withdrew patients |
|  | 2.4 If Y/PY to 2.3: Were these deviations likely to have affected the outcome? | NA |  |
|  | 2.5. If Y/PY/NI to 2.4: Were these deviations from intended intervention balanced between groups? | NA |  |
|  | 2.6 Was an appropriate analysis used to estimate the effect of assignment to intervention? | Y | Full analysis data set |
|  | 2.7 If N/PN/NI to 2.6: Was there potential for a substantial impact (on the result) of the failure to analyse participants in the group to which they were randomized? | NA |  |
|  | **Risk of bias judgement** | **Low** |  |
| **Bias due to missing outcome data** | 3.1 Were data for this outcome available for all, or nearly all, participants randomized? | Y | Only 9 patients were excluded from analysis |
|  | 3.2 If N/PN/NI to 3.1: Is there evidence that result was not biased by missing outcome data? | NA |  |
|  | 3.3 If N/PN to 3.2: Could missingness in the outcome depend on its true value? | NA |  |
|  | 3.4 If Y/PY/NI to 3.3: Is it likely that missingness in the outcome depended on its true value? | NA |  |
|  | **Risk of bias judgement** | **Low** |  |
| **Bias in measurement of the outcome** | 4.1 Was the method of measuring the outcome inappropriate? | N | The primary endpoint was time to clinical improvement whichwas defined as the time from randomization to a sustained improvement of at least one category on two consecutive days compared to the status at baseline. These measurements were  performed using a seven-category ordinal scale. |
|  | 4.2 Could measurement or ascertainment of the outcome have differed between intervention groups? | N |  |
|  | 4.3 Were outcome assessors aware of the intervention received by study participants? | Y | The study team were aware of the treatment allocation in this open-label trial. |
|  | 4.4 If Y/PY/NI to 4.3: Could assessment of the outcome have been influenced by knowledge of intervention received? | PY |  |
|  | 4.5 If Y/PY/NI to 4.4: Is it likely that assessment of the outcome was influenced by knowledge of intervention received? | PN |  |
|  | **Risk of bias judgement** | **Some concerns** |  |
| **Bias in selection of the reported result** | 5.1 Were the data that produced this result analysed in accordance with a pre-specified analysis plan that was finalized before unblinded outcome data were available for analysis? | Y | The data in protocol is same as the data reported in the paper |
|  | 5.2 ... multiple eligible outcome measurements (e.g. scales, definitions, time points) within the outcome domain? | PN |  |
|  | 5.3 ... multiple eligible analyses of the data? | PN |  |
|  | **Risk of bias judgement** | **Low** |  |
| **Overall bias** | **Risk of bias judgement** | **Some concerns** | Open label trial |

Table S5: Risk of bias assessment details of Karoyli et al. 2022, using ROB2.

| **Domain** | **Signalling question** | **Response** | **Comments** |
| --- | --- | --- | --- |
| **Bias arising from the randomization process** | 1.1 Was the allocation sequence random? | Y | All participants underwent a two-step randomization |
|  | 1.2 Was the allocation sequence concealed until participants were enrolled and assigned to interventions? | Y |  |
|  | 1.3 Did baseline differences between intervention groups suggest a problem with the randomization process? | PN | Minimal baseline differences |
|  | **Risk of bias judgement** | **Low** |  |
| **Bias due to deviations from intended interventions** | 2.1.Were participants aware of their assigned intervention during the trial? | N | This was a double-blinded trial |
|  | 2.2.Were carers and people delivering the interventions aware of participants' assigned intervention during the trial? | N |  |
|  | 2.3. If Y/PY/NI to 2.1 or 2.2: Were there deviations from the intended intervention that arose because of the experimental context? | NA |  |
|  | 2.4 If Y/PY to 2.3: Were these deviations likely to have affected the outcome? | NA |  |
|  | 2.5. If Y/PY/NI to 2.4: Were these deviations from intended intervention balanced between groups? | NA |  |
|  | 2.6 Was an appropriate analysis used to estimate the effect of assignment to intervention? | Y |  |
|  | 2.7 If N/PN/NI to 2.6: Was there potential for a substantial impact (on the result) of the failure to analyse participants in the group to which they were randomized? | NA |  |
|  | **Risk of bias judgement** | **Low** |  |
| **Bias due to missing outcome data** | 3.1 Were data for this outcome available for all, or nearly all, participants randomized? | Y | Minimal attrition |
|  | 3.2 If N/PN/NI to 3.1: Is there evidence that result was not biased by missing outcome data? | NA |  |
|  | 3.3 If N/PN to 3.2: Could missingness in the outcome depend on its true value? | NA |  |
|  | 3.4 If Y/PY/NI to 3.3: Is it likely that missingness in the outcome depended on its true value? | NA |  |
|  | **Risk of bias judgement** | **Low** |  |
| **Bias in measurement of the outcome** | 4.1 Was the method of measuring the outcome inappropriate? | N | Quantitative NP SARS-CoV-2 RNA levels were determined for viral load |
|  | 4.2 Could measurement or ascertainment of the outcome have differed between intervention groups? | PN |  |
|  | 4.3 Were outcome assessors aware of the intervention received by study participants? | N |  |
|  | 4.4 If Y/PY/NI to 4.3: Could assessment of the outcome have been influenced by knowledge of intervention received? | NA |  |
|  | 4.5 If Y/PY/NI to 4.4: Is it likely that assessment of the outcome was influenced by knowledge of intervention received? | NA |  |
|  | **Risk of bias judgement** | **Low** |  |
| **Bias in selection of the reported result** | 5.1 Were the data that produced this result analysed in accordance with a pre-specified analysis plan that was finalized before unblinded outcome data were available for analysis? | Y |  |
|  | 5.2 ... multiple eligible outcome measurements (e.g. scales, definitions, time points) within the outcome domain? | PN |  |
|  | 5.3 ... multiple eligible analyses of the data? | PN |  |
|  | **Risk of bias judgement** | **Low** | Data was analysed according to a pre specified plan. |
| **Overall bias** | **Risk of bias judgement** | **Low** |  |

Table S6: Risk of bias assessment details of Jilg et al. 2022, using ROB2.

| **Domain** | **Signalling question** | **Response** | **Comments** |
| --- | --- | --- | --- |
| **Bias arising from the randomization process** | 1.1 Was the allocation sequence random? | Y | This was a randomized, double-blinded, placebo-controlled trial |
|  | 1.2 Was the allocation sequence concealed until participants were enrolled and assigned to interventions? | Y |  |
|  | 1.3 Did baseline differences between intervention groups suggest a problem with the randomization process? | PN | Minimal baseline differences |
|  | **Risk of bias judgement** | **Low** |  |
| **Bias due to deviations from intended interventions** | 2.1.Were participants aware of their assigned intervention during the trial? | N | Participants were not aware of their assigned intervention |
|  | 2.2.Were carers and people delivering the interventions aware of participants' assigned intervention during the trial? | PN |  |
|  | 2.3. If Y/PY/NI to 2.1 or 2.2: Were there deviations from the intended intervention that arose because of the experimental context? | NA |  |
|  | 2.4 If Y/PY to 2.3: Were these deviations likely to have affected the outcome? | NA |  |
|  | 2.5. If Y/PY/NI to 2.4: Were these deviations from intended intervention balanced between groups? | NA |  |
|  | 2.6 Was an appropriate analysis used to estimate the effect of assignment to intervention? | Y |  |
|  | 2.7 If N/PN/NI to 2.6: Was there potential for a substantial impact (on the result) of the failure to analyse participants in the group to which they were randomized? | NA |  |
|  | **Risk of bias judgement** | **Low** |  |
| **Bias due to missing outcome data** | 3.1 Were data for this outcome available for all, or nearly all, participants randomized? | PY |  |
|  | 3.2 If N/PN/NI to 3.1: Is there evidence that result was not biased by missing outcome data? | NA |  |
|  | 3.3 If N/PN to 3.2: Could missingness in the outcome depend on its true value? | NA |  |
|  | 3.4 If Y/PY/NI to 3.3: Is it likely that missingness in the outcome depended on its true value? | NA |  |
|  | **Risk of bias judgement** | **Low** | Attrition was low |
| **Bias in measurement of the outcome** | 4.1 Was the method of measuring the outcome inappropriate? | N |  |
|  | 4.2 Could measurement or ascertainment of the outcome have differed between intervention groups? | N |  |
|  | 4.3 Were outcome assessors aware of the intervention received by study participants? | Y | Outcome assessors were aware of the intervention |
|  | 4.4 If Y/PY/NI to 4.3: Could assessment of the outcome have been influenced by knowledge of intervention received? | PY |  |
|  | 4.5 If Y/PY/NI to 4.4: Is it likely that assessment of the outcome was influenced by knowledge of intervention received? | PN |  |
|  | **Risk of bias judgement** | **Some concerns** |  |
| **Bias in selection of the reported result** | 5.1 Were the data that produced this result analysed in accordance with a pre-specified analysis plan that was finalized before unblinded outcome data were available for analysis? | Y |  |
|  | 5.2 ... multiple eligible outcome measurements (e.g. scales, definitions, time points) within the outcome domain? | N |  |
|  | 5.3 ... multiple eligible analyses of the data? | N |  |
|  | **Risk of bias judgement** | **Low** | Data was analysed according to a pre-specified analysis plan. |
| **Overall bias** | **Risk of bias judgement** | **Some concerns** |  |

Table S7: Risk of bias assessment details of Kim et al. 2023, using ROB2.

| **Domain** | **Signalling question** | **Response** | **Comments** |
| --- | --- | --- | --- |
| **Bias arising from the randomization process** | 1.1 Was the allocation sequence random? | Y | Subjects were randomly allocated using a computer generated list in a 2:1 ratio to treatment and control groups. Both therapies were visually identical to ensure concealment. |
|  | 1.2 Was the allocation sequence concealed until participants were enrolled and assigned to interventions? | Y |  |
|  | 1.3 Did baseline differences between intervention groups suggest a problem with the randomization process? | N |  |
|  | **Risk of bias judgement** | **Low** |  |
| **Bias due to deviations from intended interventions** | 2.1.Were participants aware of their assigned intervention during the trial? | N | Participants were unaware of their assigned intervention |
|  | 2.2.Were carers and people delivering the interventions aware of participants' assigned intervention during the trial? | PN |  |
|  | 2.3. If Y/PY/NI to 2.1 or 2.2: Were there deviations from the intended intervention that arose because of the experimental context? | NA |  |
|  | 2.4 If Y/PY to 2.3: Were these deviations likely to have affected the outcome? | NA |  |
|  | 2.5. If Y/PY/NI to 2.4: Were these deviations from intended intervention balanced between groups? | NA |  |
|  | 2.6 Was an appropriate analysis used to estimate the effect of assignment to intervention? | Y |  |
|  | 2.7 If N/PN/NI to 2.6: Was there potential for a substantial impact (on the result) of the failure to analyse participants in the group to which they were randomized? | NA |  |
|  | **Risk of bias judgement** | **Low** | There was minimal deviation from intended interventions |
| **Bias due to missing outcome data** | 3.1 Were data for this outcome available for all, or nearly all, participants randomized? | Y |  |
|  | 3.2 If N/PN/NI to 3.1: Is there evidence that result was not biased by missing outcome data? | NA |  |
|  | 3.3 If N/PN to 3.2: Could missingness in the outcome depend on its true value? | NA |  |
|  | 3.4 If Y/PY/NI to 3.3: Is it likely that missingness in the outcome depended on its true value? | NA |  |
|  | **Risk of bias judgement** | **Low** | Attrition was low |
| **Bias in measurement of the outcome** | 4.1 Was the method of measuring the outcome inappropriate? | N | The primary end point to assess drug efficacy was a change in  the shedding of the SARS-CoV-2 virus as measured by Ct obtained  from nasopharyngeal swabs on days 1 and 5 |
|  | 4.2 Could measurement or ascertainment of the outcome have differed between intervention groups? | N |  |
|  | 4.3 Were outcome assessors aware of the intervention received by study participants? | Y |  |
|  | 4.4 If Y/PY/NI to 4.3: Could assessment of the outcome have been influenced by knowledge of intervention received? | N |  |
|  | 4.5 If Y/PY/NI to 4.4: Is it likely that assessment of the outcome was influenced by knowledge of intervention received? | NA |  |
|  | **Risk of bias judgement** | **Low** | The method of measuring the outcomes was appropriate, and no difference between the groups were detected |
| **Bias in selection of the reported result** | 5.1 Were the data that produced this result analysed in accordance with a pre-specified analysis plan that was finalized before unblinded outcome data were available for analysis? | Y |  |
|  | 5.2 ... multiple eligible outcome measurements (e.g. scales, definitions, time points) within the outcome domain? | N |  |
|  | 5.3 ... multiple eligible analyses of the data? | N |  |
|  | **Risk of bias judgement** | **Low** | Data were analysed according to a pre-specified analysis plan. |
| **Overall bias** | **Risk of bias judgement** | **Low** |  |

Table S8: Risk of bias assessment details of Toback et al. 2022, using ROB2.

| **Domain** | **Signalling question** | **Response** | **Comments** |
| --- | --- | --- | --- |
| **Bias arising from the randomization process** | 1.1 Was the allocation sequence random? | Y | Participants were enrolled at the clinical department in the trial sites and randomly assigned in a 2:1 ratio to receive either camostat mesilate or placebo. All trial personnel were blinded to randomization. |
|  | 1.2 Was the allocation sequence concealed until participants were enrolled and assigned to interventions? | PY |  |
|  | 1.3 Did baseline differences between intervention groups suggest a problem with the randomization process? | N |  |
|  | **Risk of bias judgement** | **Low** |  |
| **Bias due to deviations from intended interventions** | 2.1.Were participants aware of their assigned intervention during the trial? | N | Participants were not aware of their assigned intervention |
|  | 2.2.Were carers and people delivering the interventions aware of participants' assigned intervention during the trial? | N |  |
|  | 2.3. If Y/PY/NI to 2.1 or 2.2: Were there deviations from the intended intervention that arose because of the experimental context? | NA |  |
|  | 2.4 If Y/PY to 2.3: Were these deviations likely to have affected the outcome? | NA |  |
|  | 2.5. If Y/PY/NI to 2.4: Were these deviations from intended intervention balanced between groups? | NA |  |
|  | 2.6 Was an appropriate analysis used to estimate the effect of assignment to intervention? | Y | Modified ITT |
|  | 2.7 If N/PN/NI to 2.6: Was there potential for a substantial impact (on the result) of the failure to analyse participants in the group to which they were randomized? | NA |  |
|  | **Risk of bias judgement** | **Low** |  |
| **Bias due to missing outcome data** | 3.1 Were data for this outcome available for all, or nearly all, participants randomized? | Y | Minimal missing data |
|  | 3.2 If N/PN/NI to 3.1: Is there evidence that result was not biased by missing outcome data? | NA |  |
|  | 3.3 If N/PN to 3.2: Could missingness in the outcome depend on its true value? | NA |  |
|  | 3.4 If Y/PY/NI to 3.3: Is it likely that missingness in the outcome depended on its true value? | NA |  |
|  | **Risk of bias judgement** | **Low** | Attrition was low |
| **Bias in measurement of the outcome** | 4.1 Was the method of measuring the outcome inappropriate? | N | The primary endpoint was time to clinical improvement, defined as live hospital discharge or an improvement of at least 2 points from baseline on the 7-point ordinal scale, which ever came first |
|  | 4.2 Could measurement or ascertainment of the outcome have differed between intervention groups? | N |  |
|  | 4.3 Were outcome assessors aware of the intervention received by study participants? | PN |  |
|  | 4.4 If Y/PY/NI to 4.3: Could assessment of the outcome have been influenced by knowledge of intervention received? | NA |  |
|  | 4.5 If Y/PY/NI to 4.4: Is it likely that assessment of the outcome was influenced by knowledge of intervention received? | NA |  |
|  | **Risk of bias judgement** | **Low** | The method of measuring the outcomes was appropriate, and no difference between the groups were detected |
| **Bias in selection of the reported result** | 5.1 Were the data that produced this result analysed in accordance with a pre-specified analysis plan that was finalized before unblinded outcome data were available for analysis? | Y |  |
|  | 5.2 ... multiple eligible outcome measurements (e.g. scales, definitions, time points) within the outcome domain? | PN |  |
|  | 5.3 ... multiple eligible analyses of the data? | PN |  |
|  | **Risk of bias judgement** | **Low** | Data were analysed according to a pre-specified analysis plan. |
| **Overall bias** | **Risk of bias judgement** | **Low** | Low risk domains |

Table S9: Risk of bias assessment details of Gunst et al. 2021, using ROB2.

| **Domain** | **Signalling question** | **Response** | **Comments** |
| --- | --- | --- | --- |
| **Bias arising from the randomization process** | 1.1 Was the allocation sequence random? | Y | Randomization was performed using a minimization method |
|  | 1.2 Was the allocation sequence concealed until participants were enrolled and assigned to interventions? | Y |  |
|  | 1.3 Did baseline differences between intervention groups suggest a problem with the randomization process? | N |  |
|  | **Risk of bias judgement** | **Low** |  |
| **Bias due to deviations from intended interventions** | 2.1.Were participants aware of their assigned intervention during the trial? | N | Patients, investigators/subinvestigators, study coordinators, and other study personnel were blinded throughout the study |
|  | 2.2.Were carers and people delivering the interventions aware of participants' assigned intervention during the trial? | N |  |
|  | 2.3. If Y/PY/NI to 2.1 or 2.2: Were there deviations from the intended intervention that arose because of the experimental context? | NA |  |
|  | 2.4 If Y/PY to 2.3: Were these deviations likely to have affected the outcome? | NA |  |
|  | 2.5. If Y/PY/NI to 2.4: Were these deviations from intended intervention balanced between groups? | NA |  |
|  | 2.6 Was an appropriate analysis used to estimate the effect of assignment to intervention? | Y | Modified ITT |
|  | 2.7 If N/PN/NI to 2.6: Was there potential for a substantial impact (on the result) of the failure to analyse participants in the group to which they were randomized? | NA |  |
|  | **Risk of bias judgement** | **Low** |  |
| **Bias due to missing outcome data** | 3.1 Were data for this outcome available for all, or nearly all, participants randomized? | Y | Minimal missing data |
|  | 3.2 If N/PN/NI to 3.1: Is there evidence that result was not biased by missing outcome data? | NA |  |
|  | 3.3 If N/PN to 3.2: Could missingness in the outcome depend on its true value? | NA |  |
|  | 3.4 If Y/PY/NI to 3.3: Is it likely that missingness in the outcome depended on its true value? | NA |  |
|  | **Risk of bias judgement** | **Low** |  |
| **Bias in measurement of the outcome** | 4.1 Was the method of measuring the outcome inappropriate? | N | The primary efficacy endpoint was the time to the frst two consecutive negative SARS-CoV-2 tests performed at the hospital’s local laboratory |
|  | 4.2 Could measurement or ascertainment of the outcome have differed between intervention groups? | N |  |
|  | 4.3 Were outcome assessors aware of the intervention received by study participants? | N |  |
|  | 4.4 If Y/PY/NI to 4.3: Could assessment of the outcome have been influenced by knowledge of intervention received? | NA |  |
|  | 4.5 If Y/PY/NI to 4.4: Is it likely that assessment of the outcome was influenced by knowledge of intervention received? | NA |  |
|  | **Risk of bias judgement** | **Low** | The method of measuring the outcomes was appropriate. |
| **Bias in selection of the reported result** | 5.1 Were the data that produced this result analysed in accordance with a pre-specified analysis plan that was finalized before unblinded outcome data were available for analysis? | Y |  |
|  | 5.2 ... multiple eligible outcome measurements (e.g. scales, definitions, time points) within the outcome domain? | N |  |
|  | 5.3 ... multiple eligible analyses of the data? | N |  |
|  | **Risk of bias judgement** | **Low** | Data were analysed according to a pre-specified analysis plan. |
| **Overall bias** | **Risk of bias judgement** | **Low** |  |

Table S10: Risk of bias assessment details of Kinoshita et al. 2022, using ROB2.

| **Domain** | **Signalling question** | **Response** | **Comments** |
| --- | --- | --- | --- |
| **Bias arising from the randomization process** | 1.1 Was the allocation sequence random? | PY | Participants were to be randomized in a 2:1 ratio of camostat:placebo. |
|  | 1.2 Was the allocation sequence concealed until participants were enrolled and assigned to interventions? | PY |  |
|  | 1.3 Did baseline differences between intervention groups suggest a problem with the randomization process? | N | Minimal baseline differences |
|  | **Risk of bias judgement** | **Low** |  |
| **Bias due to deviations from intended interventions** | 2.1.Were participants aware of their assigned intervention during the trial? | N | Participants and care-givers were masked during the trial |
|  | 2.2.Were carers and people delivering the interventions aware of participants' assigned intervention during the trial? | N |  |
|  | 2.3. If Y/PY/NI to 2.1 or 2.2: Were there deviations from the intended intervention that arose because of the experimental context? | NA |  |
|  | 2.4 If Y/PY to 2.3: Were these deviations likely to have affected the outcome? | NA |  |
|  | 2.5. If Y/PY/NI to 2.4: Were these deviations from intended intervention balanced between groups? | NA |  |
|  | 2.6 Was an appropriate analysis used to estimate the effect of assignment to intervention? | Y |  |
|  | 2.7 If N/PN/NI to 2.6: Was there potential for a substantial impact (on the result) of the failure to analyse participants in the group to which they were randomized? | NA |  |
|  | **Risk of bias judgement** | **Low** |  |
| **Bias due to missing outcome data** | 3.1 Were data for this outcome available for all, or nearly all, participants randomized? | PY | Minimal attrition |
|  | 3.2 If N/PN/NI to 3.1: Is there evidence that result was not biased by missing outcome data? | NA |  |
|  | 3.3 If N/PN to 3.2: Could missingness in the outcome depend on its true value? | NA |  |
|  | 3.4 If Y/PY/NI to 3.3: Is it likely that missingness in the outcome depended on its true value? | NA |  |
|  | **Risk of bias judgement** | **Low** |  |
| **Bias in measurement of the outcome** | 4.1 Was the method of measuring the outcome inappropriate? | N | Outcome was measured as time (in days) from initiation of study treatment until normalization of fever (≤ 37.2 °C oral or tympanic) and sustained for at least 3 days only assessed in participants who experienced a fever within 1 day of enrollment up to Day 28. |
|  | 4.2 Could measurement or ascertainment of the outcome have differed between intervention groups? | N |  |
|  | 4.3 Were outcome assessors aware of the intervention received by study participants? | N |  |
|  | 4.4 If Y/PY/NI to 4.3: Could assessment of the outcome have been influenced by knowledge of intervention received? | PN |  |
|  | 4.5 If Y/PY/NI to 4.4: Is it likely that assessment of the outcome was influenced by knowledge of intervention received? | NA |  |
|  | **Risk of bias judgement** | **Low** | The method of measuring the outcomes was appropriate, and no difference between the groups were detected |
| **Bias in selection of the reported result** | 5.1 Were the data that produced this result analysed in accordance with a pre-specified analysis plan that was finalized before unblinded outcome data were available for analysis? | NI |  |
|  | 5.2 ... multiple eligible outcome measurements (e.g. scales, definitions, time points) within the outcome domain? | PN |  |
|  | 5.3 ... multiple eligible analyses of the data? | PN |  |
|  | **Risk of bias judgement** | **Some concerns** | Protocol did not specify a set analysis plan |
| **Overall bias** | **Risk of bias judgement** | **Some concerns** |  |

Table S11: Risk of bias assessment details of NCT04583592, using ROB2.

| **Domain** | **Signalling question** | **Response** | **Comments** |
| --- | --- | --- | --- |
| **Bias arising from the randomization process** | 1.1 Was the allocation sequence random? | Y | Study plan included randomization with double masking |
|  | 1.2 Was the allocation sequence concealed until participants were enrolled and assigned to interventions? | PY |  |
|  | 1.3 Did baseline differences between intervention groups suggest a problem with the randomization process? | N |  |
|  | **Risk of bias judgement** | **Low** |  |
| **Bias due to deviations from intended interventions** | 2.1.Were participants aware of their assigned intervention during the trial? | N | Double masking was performed |
|  | 2.2.Were carers and people delivering the interventions aware of participants' assigned intervention during the trial? | N |  |
|  | 2.3. If Y/PY/NI to 2.1 or 2.2: Were there deviations from the intended intervention that arose because of the experimental context? | NA |  |
|  | 2.4 If Y/PY to 2.3: Were these deviations likely to have affected the outcome? | NA |  |
|  | 2.5. If Y/PY/NI to 2.4: Were these deviations from intended intervention balanced between groups? | NA |  |
|  | 2.6 Was an appropriate analysis used to estimate the effect of assignment to intervention? | Y |  |
|  | 2.7 If N/PN/NI to 2.6: Was there potential for a substantial impact (on the result) of the failure to analyse participants in the group to which they were randomized? | NA |  |
|  | **Risk of bias judgement** | **Low** |  |
| **Bias due to missing outcome data** | 3.1 Were data for this outcome available for all, or nearly all, participants randomized? | NI |  |
|  | 3.2 If N/PN/NI to 3.1: Is there evidence that result was not biased by missing outcome data? | PY |  |
|  | 3.3 If N/PN to 3.2: Could missingness in the outcome depend on its true value? | NA |  |
|  | 3.4 If Y/PY/NI to 3.3: Is it likely that missingness in the outcome depended on its true value? | NA |  |
|  | **Risk of bias judgement** | **Low** |  |
| **Bias in measurement of the outcome** | 4.1 Was the method of measuring the outcome inappropriate? | N | Time Until Resolution of Symptoms was defined as absence of moderate or severe symptoms for at least 24 hours for those reporting moderate or severe symptoms at baseline. |
|  | 4.2 Could measurement or ascertainment of the outcome have differed between intervention groups? | N |  |
|  | 4.3 Were outcome assessors aware of the intervention received by study participants? | Y |  |
|  | 4.4 If Y/PY/NI to 4.3: Could assessment of the outcome have been influenced by knowledge of intervention received? | PN |  |
|  | 4.5 If Y/PY/NI to 4.4: Is it likely that assessment of the outcome was influenced by knowledge of intervention received? | NA |  |
|  | **Risk of bias judgement** | **Low** | The method of measuring the outcomes was appropriate. |
| **Bias in selection of the reported result** | 5.1 Were the data that produced this result analysed in accordance with a pre-specified analysis plan that was finalized before unblinded outcome data were available for analysis? | NI | No pre-specified plan |
|  | 5.2 ... multiple eligible outcome measurements (e.g. scales, definitions, time points) within the outcome domain? | N |  |
|  | 5.3 ... multiple eligible analyses of the data? | N |  |
|  | **Risk of bias judgement** | **Some concerns** |  |
| **Overall bias** | **Risk of bias judgement** | **Some concerns** |  |

Table S12: Risk of bias assessment details of NCT04524663, using ROB2.

| Outcome | No. of  participants (/) | No. of  trials | Quantitative data synthesis | | | | | Heterogeneity analysis | | |
| --- | --- | --- | --- | --- | --- | --- | --- | --- | --- | --- |
|  |  |  | RR/  MD | 95% CI | | Z value | p-value | df | p-value | I2 (%) |
| **Time to symptom improvement** | | | | | | | | | | |
| All studies | 945 | 4 | -0.09 | -0.56, 0.38 | | 0.38 | 0.70 | 3 | <0.00001 | 99 |
| Omitting  Gunst et al | 740 | 3 | 0.03 | -0.46, 0.52 | | 0.12 | 0.91 | 2 | <0.00001 | 99 |
| Omitting  Karolyi et al | 744 | 3 | -0.84 | -1.31, -0.36 | | 3.46 | 000.5 | 2 | 0.88 | 0 |
| Omitting  Kim et al | 622 | 3 | 0.52 | -0.12, 1.16 | | 1.60 | 0.11 | 2 | <0.00001 | 99 |
| Omitting  Jilg et al | 729 | 3 | 0.40 | -0.21, 1.01 | | 1.29 | 0.20 | 2 | <0.00001 | 99 |
| **ICU admission or Mechanical ventilation** | | | | | | | | | | |
| All studies | 559 | 3 | 0.55 | 0.20, 1.53 | | 1.15 | 0.25 | 1 | 0.13 | 57 |
| Omitting  Gunst et al | 354 | 2 | 0.30 | 0.10, 0.90 | | 2.25 | 0.03 | NA | NA | NA |
| Omitting  Karolyi et al | 358 | 2 | 0.87 | 0.38, 1.97 | | 0.34 | 0.74 | NA | NA | NA |
| Omitting  Kinoshita et al | 406 | 2 | 0.55 | 0.20, 1.53 | | 1.15 | 0.25 | 1 | 0.13 | 57 |
| **Any adverse events** | | | | | | | | | | |
| All studies | 1226 | 8 | 0.93 | 0.67, 1.29 | | 0.44 | 0.66 | 7 | <0.00001 | 80 |
| Omitting  Gunst et al | 1073 | 7 | 0.84 | 0.59, 1.20 | | 0.96 | 0.34 | 6 | <0.0001 | 80 |
| Omitting  Kinoshita et al | 1073 | 7 | 0.95 | 0.65, 1.37 | | 0.30 | 0.77 | 6 | <0.0001 | 82 |
| Omitting  Tobback et al | 1136 | 7 | 0.87 | 0.59, 1.28 | | 0.70 | 0.48 | 6 | <0.0002 | 77 |
| Omitting  Chupp et al | 1156 | 7 | 0.88 | 0.62, 1.26 | | 0.70 | 0.48 | 6 | <0.00001 | 82 |
| Omitting  Karolyi et al | 1025 | 7 | 1.03 | 0.76, 1.40 | | 0.18 | 0.86 | 6 | 0.003 | 70 |
| Omitting  NCT04524663 | 1177 | 7 | 1.01 | 0.75, 1.35 | | 0.06 | 0.96 | 6 | 0.0003 | 76 |
| Omitting  NCT04583592 | 932 | 7 | 0.96 | 0.68, 1.36 | | 0.21 | 0.84 | 6 | <0.0001 | 81 |
| Omitting  Jilg et al | 1010 | 7 | 0.89 | 0.60, 1.32 | | 0.60 | 0.55 | 6 | <0.00001 | 83 |
| **ICU admission or Mechanical ventilation** | | | | | | | | | | |
| All studies | 559 | 3 | 0.55 | | 0.20, 1.53 | 1.15 | 0.25 | 1 | 0.13 | 57 |
| Omitting  Gunst et al | 354 | 2 | 0.30 | | 0.10, 0.90 | 2.25 | 0.03 | NA | NA | NA |
| Omitting  Karolyi et al | 358 | 2 | 0.87 | | 0.38, 1.97 | 0.34 | 0.74 | NA | NA | NA |
| Omitting  Kinoshita et al | 406 | 2 | 0.55 | | 0.20, 1.53 | 1.15 | 0.25 | 1 | 0.13 | 57 |

***Table 13,*** *Sensitivity Analysis*
